# Capture Hi-C identifies a novel causal gene, *IL20RA*, in the pan-autoimmune genetic susceptibility region 6q23

**DOI:** 10.1186/s13059-016-1078-x

**Published:** 2016-11-01

**Authors:** Amanda McGovern, Stefan Schoenfelder, Paul Martin, Jonathan Massey, Kate Duffus, Darren Plant, Annie Yarwood, Arthur G. Pratt, Amy E. Anderson, John D. Isaacs, Julie Diboll, Nishanthi Thalayasingam, Caroline Ospelt, Anne Barton, Jane Worthington, Peter Fraser, Stephen Eyre, Gisela Orozco

**Affiliations:** 1 Arthritis Research UK Centre for Genetics and Genomics, Division of Musculoskeletal and Dermatological Sciences, School of Biological Sciences, Faculty of Biology, Medicine and Health, Manchester Academic Health Science Centre, The University of Manchester, Stopford Building, Oxford Road, Manchester, M13 9PT UK; 2 Nuclear Dynamics Programme, The Babraham Institute, Cambridge, CB22 3AT UK; 3 NIHR Manchester Musculoskeletal BRU, Manchester Academic Health Sciences Centre, Central Manchester Foundation Trust, Manchester, UK; 4 Institute of Cellular Medicine (Musculoskeletal Research Group), Newcastle University, Newcastle upon Tyne, NE2 4HH UK; 5 Center of Experimental Rheumatology Department of Rheumatology, University Hospital of Zurich, Wagistrasse 14, 8952 Schlieren, Switzerland

**Keywords:** Autoimmunity, Single nucleotide polymorphisms (SNP), Genome-wide association studies (GWAS), Causal genes, Functional genomics, Capture Hi-C

## Abstract

**Background:**

The identification of causal genes from genome-wide association studies (GWAS) is the next important step for the translation of genetic findings into biologically meaningful mechanisms of disease and potential therapeutic targets. Using novel chromatin interaction detection techniques and allele specific assays in T and B cell lines, we provide compelling evidence that redefines causal genes at the 6q23 locus, one of the most important loci that confers autoimmunity risk.

**Results:**

Although the function of disease-associated non-coding single nucleotide polymorphisms (SNPs) at 6q23 is unknown, the association is generally assigned to *TNFAIP3*, the closest gene. However, the DNA fragment containing the associated SNPs interacts through chromatin looping not only with *TNFAIP3*, but also with *IL20RA*, located 680 kb upstream. The risk allele of the most likely causal SNP, rs6927172, is correlated with both a higher frequency of interactions and increased expression of *IL20RA*, along with a stronger binding of both the NFκB transcription factor and chromatin marks characteristic of active enhancers in T-cells.

**Conclusions:**

Our results highlight the importance of gene assignment for translating GWAS findings into biologically meaningful mechanisms of disease and potential therapeutic targets; indeed, monoclonal antibody therapy targeting IL-20 is effective in the treatment of rheumatoid arthritis and psoriasis, both with strong GWAS associations to this region.

**Electronic supplementary material:**

The online version of this article (doi:10.1186/s13059-016-1078-x) contains supplementary material, which is available to authorized users.

## Background

In recent years, understanding of the genetic predisposition to human complex diseases has been dramatically enhanced through the application of well-powered genome-wide association studies (GWAS). Thousands of genetic variants (single nucleotide polymorphisms or SNPs) have been associated with disease [1], but the functional role of the vast majority of these disease variants is yet to be explored. This is due to the fact that around 90 % lie outside known coding regions of the genome and, therefore, their potential role in pathological mechanisms is not obvious [2, 3]. There is now strong evidence supporting a role for these non-coding variants in transcriptional regulation as they are enriched in cell type and stimulus-specific enhancer regions [4–6], which are capable of influencing their target genes through long-range chromosomal interactions [7–10]. Traditionally, GWAS associated variants have been annotated with the closest or most biologically relevant candidate gene within arbitrarily defined distances. However, this approach has been challenged by recent chromatin looping interaction studies showing that interactions between enhancers and their target genes can occur over unexpectedly large genetic distances, often bypassing the nearest genes [11–13].

In order to link GWAS associated variants with disease-causing genes, we have employed a hypothesis-free method that enables the targeted characterisation of chromatin interactions at the genome-wide level at high resolution. While chromosome conformation capture studies utilising chromosome conformation capture (3C), chromosome conformation capture-on-chip (4C) and chromosome conformation capture carbon copy (5C) have been successfully used to identify interactions between regulatory elements and target genes [14–16], regions of interest and potential targets have to be considered a priori. By contrast, Hi-C allows interrogation of all interactions on a genome-wide scale [17], but the approach lacks resolution. Recently, a new method that incorporates a targeted sequence capture step into Hi-C, Capture Hi-C (CHi-C), has been developed [13, 18–20]. The method has facilitated the identification of interactions between non-coding SNPs associated with cancer and autoimmunity with their targets [18, 19, 21].

**Fig. 1:**
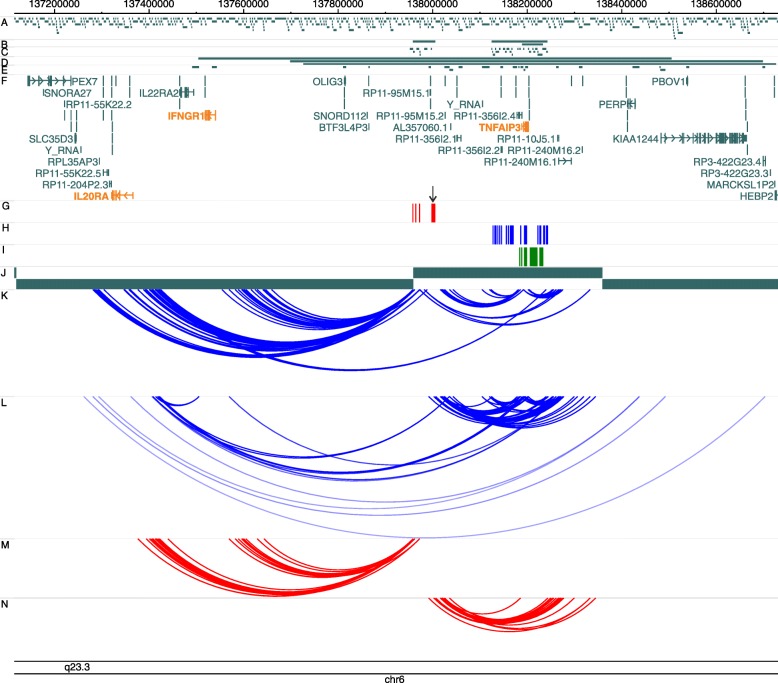
Long-range interactions in the 6q23 locus. Genomic co-ordinates are shown along the *top* of each *panel* and *tracks* are labelled **a**–**n**. **a** HindIII restriction fragments. **b**–**e** Regions targeted and restriction fragments included in the Region (**b**, **c**) and Promoter (**d**, **e**) Capture experiments. **f** GENCODE V17 genes. **g**–**i** 1000 Genomes SNPs in LD (r2 ≥ 0.8) with the index SNPs rs6920220, associated with RA, SLE, celiac disease, T1D and IBD (**g**), rs7752903, associated with RA, SLE and celiac disease (**h**) and rs610604, associated with Ps and PsA (**i**). **j** Topologically associated domains (TADs) in GM12878 cells [20]. **k**–**n** Significant interactions identified in the Region and Promoter capture experiments in GM12878 (**k**, **l**) and Jurkat (**m**, **n**) cells. The *black arrow* indicates the position of the rs6927172 SNP

The chromosomal region 6q23 contains several variants associated with many autoimmune diseases. These associations have been annotated to the *TNFAIP3* gene, the closest most plausible causal gene within the locus, with independent variants within the gene associated with different diseases. There are three distinct linkage disequilibrium (LD) blocks independently associated with a range of autoimmune diseases, including rheumatoid arthritis (RA), systemic lupus erythematosus (SLE), celiac disease (CeD), type 1 diabetes (T1D), inflammatory bowel disease (IBD), psoriasis (Ps) and psoriatic arthritis (PsA) [22–29]. One region, containing SNPs associated with RA, SLE, CeD, IBD and T1D, tagged by the rs6920220 SNP, lies a considerable distance (>181 kb) from the *TNFAIP3* gene and its functional role has, so far, been underexplored (Fig. 1g). The second, independent association signal, tagged by rs7752903, and predisposing to RA, SLE and CeD, spans around 100 kb and includes the *TNFAIP3* gene (Fig. 1h). There is evidence that a TT > A polymorphism located within this LD block, 42 kb downstream of *TNFAIP3*, alters A20 (the protein encoded by *TNFAIP3*) expression through impaired delivery of NFκB to the *TNFAIP3* promoter [9, 30, 31]. An additional association signal, tagged by rs610604, confers risk to Ps and PsA (Fig. 1i).

The aim of the current work was to identify causal disease genes and refine the likely causal SNPs at the autoimmunity locus 6q23 by studying long-range chromatin interactions using CHi-C, to validate findings using genotype specific 3C and augment the evidence further with cell-type and genotype specific expression quantitative trait loci (eQTL) and chromatin immunoprecipitation (ChIP) analysis. Here, we report a new causal candidate disease gene within the 6q23 region, *IL20RA*, which encodes one of the subunits of the receptor for the pro-inflammatory cytokine IL-20. Our results suggest that non-coding SNPs associated with RA, SLE, CeD, IBD and T1D alter a regulatory element of *IL20RA*, some 680 kb away, which acts through long-range interactions with the *IL20RA* promoter, resulting in increased expression of the gene.

## Results

### 6q23 variants interact with several genes, including *IL20RA*, through chromatin looping

Investigation of chromatin interactions at the 6q23 locus was carried out as part of a larger study that included all known risk loci for RA, JIA, PsA and T1D [21]. We selected four target regions mapping to 6q23 for enrichment in two different CHi-C experiments: first, the Region Capture Hi-C targeted the LD blocks (r^2^ > 0.8) for three SNPs associated with the diseases under study: rs6920220 (RA, T1D, JIA), rs7752903 (RA) and rs610604 (Ps, PsA) (Fig. 1a–c); second, the Promoter Capture targeted all known gene promoters overlapping the region 500 kb upstream and downstream of the lead disease associated SNPs (Fig. 1d and e). CHi-C libraries were generated for two cell lines: GM12878, a B-lymphoblastoid cell line, and Jurkat, a CD4+ T-lymphoblastoid cell line.

The LD block containing the intergenic 6q23 SNP, rs6920220, targeted in the region capture, spans 47.3 kb (chr6:137959235–138006504) and contains seven restriction fragments (Fig. 1b, c and g). Of these, five were involved in statistically significant interactions. This intergenic region, containing SNPs associated with multiple autoimmune diseases, demonstrated a complex pattern of interactions, shown in Fig. 1k–n. Intriguingly, these long-range interactions involved robust and compelling interactions with both *IL20RA* and *IFNGR1*, reflecting putative roles in regulating the expression of these genes. There is also evidence of interactions with the long non-coding RNAs (lncRNAs) RP11-10J5.1 and RP11-240M16.1 downstream of the *TNFAIP3* gene.

The Region Capture experiments targeting both the LD block containing RA (rs7752903) and Ps/PsA (rs610604) associated variants, and spanning the *TNFAIP3* gene along with its upstream and downstream regions (Fig. 1h and i), showed interactions with a region proximal to the rs6920220 LD block, encompassing the lncRNAs RP11-95M15.2 (a *PTPN11* pseudogene) and RP11-356I2.1, the miRNA AL357060.1 and also an upstream region containing non-coding RNAs (Y_RNA and RP11-356I2.2) (Fig. 1k). Finally, the Region Capture experiment detected an interaction involving *TNFAIP3* and a region containing the lncRNAs RP11-10J5.1 and RP11-240M16.1 approximately 50 kb downstream of the gene, which in turn, also interacts with the intergenic rs6920220-tagged LD block. Interestingly, this region, downstream of *TNFAIP3*, showed an additional long-range interaction with the *IL20RA* gene (Fig. 1k).

These interactions were independently validated in the second, separate Promoter Capture experiment (Fig. 1d, e, l and n). Furthermore, we detected an interaction between the promoters of *TNFAIP3* and *IL20RA* that was not revealed in the Region Capture experiment, as promoters were excluded from the Region Capture experiment (Fig. 1l).

**Fig. 2:**
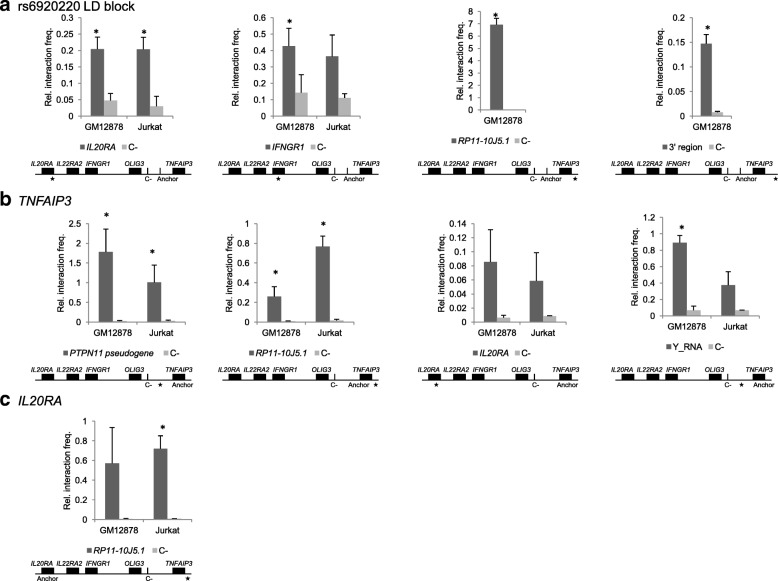
Validation of CHi-C results by 3C-qPCR in GM12878 and Jurkat cell lines. The *graphs* show the relative interaction frequency of (**a**) the 6q23 intergenic disease SNPs tagged by rs6920220, (**b**) the *TNFAIP3* gene and (**c**) the *IL20RA* gene with their respective targets (*dark grey*) compared to control, non-interacting fragments (C-, *light grey*). *Diagrams* below each *graph* show the approximate location of the primers for the anchor, negative control (C-) and target (★) regions. *Error bars* indicate standard deviation of three biological replicates; * indicates t-test *P* value <0.05

Importantly, we sought validation of CHi-C results by 3C-quantitative real-time polymerase chain reaction (qPCR). Higher interaction frequencies were confirmed for all interrogated regions, compared to adjacent non-interacting regions (Fig. 2).

To validate our analysis method, we reanalysed our CHi-C data using a recently developed analytical algorithm, CHiCAGO (Capture HiC Analysis of Genomic Organisation (http://biorxiv.org/content/early/2015/10/05/028068). The pattern of chromatin loops obtained when we applied CHiCAGO was more complex, although it confirmed our findings (Additional file 1: Figure S1). Additional interactions not passing the significance threshold in the initial analysis were found between *IL22RA2* and the rs6920220 LD block, *IL22RA2* and the RP11-10J5.1 and RP11-240M16.1 lncRNAs downstream of *TNFAIP3*, *IFNGR1* and the rs6920220 LD block and *IFNGR1* and *TNFAIP3*. Further investigations will be required to validate these interactions.

Therefore, using CHi-C and validated by 3C-qPCR, we have confirmed that an intergenic region containing SNPs associated with RA, T1D, SLE, CeD and IBD, tagged by rs6920220 interacts with *IL20RA*, *IFNGR1* and the lncRNAs RP11-10J5.1 and RP11-240M16.1. We also confirmed that a second region, containing *TNFAIP3* and SNPs associated with RA, SLE, CeD, PsA and Ps, interacts with *IL20RA*, and a number of lncRNAs, including RP11-10J5.1 and RP11-240M16.1.

### rs6927172 shows the most regulatory potential among all SNPs in LD with the top GWAS signal

Although rs6920220 is associated with a host of autoimmune diseases, its intergenic location and underexplored functional role means no causal gene has so far been unequivocally assigned. We therefore focused our work on this SNP region. The autoimmunity associated SNP rs6920220 is in tight LD (r^2^ > 0.8) with eight other SNPs (rs6933404, rs62432712, rs2327832, rs928722, rs6927172, rs35926684, rs17264332 and rs11757201). After confirmation that these SNPs are involved in long-range interactions with several genes, including *IL20RA*, *IFNGR1*, and several lncRNAs, we aimed to narrow down the most plausible causal SNP using bioinformatics. Haploreg v4.1 was used to identify SNPs with regulatory potential [32], showing that rs6927172 demonstrates a number of lines of evidence to support a function in disease causality, including mapping to an enhancer in B-lymphoblastoid cell lines, primary stimulated Th17, and T-regulatory cells (ChromHMM chromatin state). It also maps to a region of open chromatin, characterised by DNase hypersensitivity, shows evidence of binding regulatory proteins and lies in a conserved region (Table 1). Furthermore, analysis of a library of transcription factor binding site position weight matrices predicts that the SNP alters the binding site of eight transcription factors, including NFκB and BCL3 [32]. Additionally, investigation of functional annotation using RegulomeDBVersion 1.1 assigned the highest score to rs6927172 [33] (Additional file 1: Table S1). This evidence suggests that rs6927172 shows the most regulatory potential of those in LD with rs6920220. In support of this, a previous study showed evidence of differential transcription factor binding to rs6927172 alleles [34].

**Table 1:** Functional annotation of SNPs in the 6q23 intergenic LD block tagged by rs6920220 using Haploregv4.1

pos (hg19)	LD (r^2^)	Variant	Ref	Alt	AFR freq	AMR freq	ASN freq	EUR freq	SiPhy cons	Promoter histone marks	Enhancer histone marks	DNAse	Proteins bound	Motifs changed
chr6:137959235	0.89	rs6933404	T	C	0.11	0.11	0.00	0.17			Blood^a^	GI^b^		STAT
chr6:137964697	0.88	rs62432712	A	G	0.08	0.10	0.00	0.17						Pax7,RORalpha1,Vax2
chr6:137973068	0.93	rs2327832	A	G	0.11	0.11	0.00	0.17			5 tissues^c^	GI,GI,PLCNT^d^		10 altered motifs^e^
chr6:137973832	0.92	rs928722	C	T	0.11	0.11	0.00	0.17			GI, PLCNT, LIV^f^			4 altered motifs^g^
chr6:137999562	0.84	rs35926684	GA	G	0.14	0.12	0.00	0.18			BLD^h^			4 altered motifs^i^
chr6:138002175	1	rs6927172	C	G	0.12	0.10	0.00	0.17	Yes	4 tissues^j^	7 tissues^k^	14 tissues^l^	13 bound proteins^m^	8 altered motifs^n^
chr6:138003822	1	rs11757201	G	C	0.08	0.10	0.00	0.17						Mrg,Sp4
chr6:138005515	1	rs17264332	A	G	0.12	0.10	0.00	0.17			BLD^o^			5 altered motifs^p^
chr6:138006504	1	rs6920220	G	A	0.12	0.10	0.00	0.17						Hltf

The following settings were used: LD threshold, r^2^ ≥ 0.8; 1000G Phase 1 population for LD calculation: EUR; Source for epigenomes: ChromHMM (25-state model using 12 imputed marks); Mammalian conservation algorithm: SiPhy-omega

^a^Dnd41 TCellLeukemia cell line

^b^Small intestine

^c^Fetal intestine small, fetal intestine large, rectal mucosa donor 31, duodenum mucosa, small intestine, rectal mucosa donor 29, stomach mucosa, placenta, fetal muscle leg, duodenum smooth muscle, fetal stomach, sigmoid colon, colonic mucosa, fetal adrenal gland, HepG2 hepatocellular carcinoma cell line

^d^Fetal intestine large, fetal intestine small, placenta

^e^BCL, GR, HDAC2, Irf, Nanog, Pou1f1, Pou2f2, RXRA, STAT, P300

^f^Duodenum mucosa, fetal intestine large, fetal intestine small, placenta, rectal mucosa donor 31, small intestine, stomach mucosa, HepG2 hepatocellular carcinoma cell line

^g^BCL, NRSF, Smad, Whn

^h^Primary monocytes from peripheral blood, primary neutrophils from peripheral blood, primary B cells from cord blood, primary T helper cells PMA-I stimulated, primary T helper 17 cells PMA-I stimulated, GM12878 lymphoblastoid cells, monocytes-CD14+ RO01746 cells

^i^CIZ, Foxd3, HDAC2, Nanog

^j^Primary T helper naïve cells from peripheral blood, primary B cells from peripheral blood, primary natural killer cells from peripheral blood, primary hematopoietic stem cells G-CSF-mobilised female, primary hematopoietic stem cells short-term culture, adipose nuclei, duodenum smooth muscle, colon smooth muscle, rectal mucosa donor 29, stomach mucosa, duodenum mucosa, liver

^k^A549 EtOH 0.02 pct lung carcinoma cell line, HeLa-S3 cervical carcinoma cell line, primary mononuclear cells from peripheral blood, primary T cells effector/memory enriched from peripheral blood, primary T cells from cord blood, primary T regulatory cells from peripheral blood, primary T helper cells from peripheral blood, primary T helper cells PMA-I stimulated, primary T helper 17 cells PMA-I stimulated, primary T helper memory cells from peripheral blood 1, primary T helper memory cells from peripheral blood 2, primary T CD8+ memory cells from peripheral blood, primary T helper naïve cells from peripheral blood, primary T CD8+ naïve cells from peripheral blood, primary monocytes from peripheral blood, primary B cells from cord blood, primary hematopoietic stem cells, primary hematopoietic stem cells G-CSF-mobilised male, primary neutrophils from peripheral blood, bone marrow derived cultured mesenchymal stem cells, Dnd41 TCellLeukemia cell line, GM12878 lymphoblastoid cells, HUVEC umbilical vein endothelial primary cells, monocytes-CD14+ RO01746 primary cells, osteoblast primary cells, mesenchymal stem cell derived adipocyte cultured cells

^l^A549 EtOH 0.02 pct lung carcinoma cell line, HeLa-S3 cervical carcinoma cell line, primary monocytes from peripheral blood, GM12878 lymphoblastoid cells, HUVEC umbilical vein endothelial primary cells, monocytes-CD14+ RO01746 primary cells, foreskin fibroblast primary cells skin01, HSMM cell derived skeletal muscle myotubes cells, primary hematopoietic stem cells G-CSF-mobilised female, primary B cells from peripheral blood, H1 derived mesenchymal stem cells, foreskin fibroblast primary cells skin02, foreskin keratinocyte primary cells skin02, HSMM skeletal muscle myoblasts cells

^m^GR (A549), ERALPHA_A (ECC-1), IRF4 (GM12878), CFOS (HUVEC), CJUN (HUVEC), GATA2 (HUVEC), CEBPB (HeLa-S3), JUND (HeLa-S3), P300 (HeLa-S3), STAT1 (HeLa-S3), MAFK (K562), STAT3 (MCF10A-Er-Src), KAP1 (U2OS)

^n^BCL, ERalpha-a, Ets, LXR, NFkB, RORalpha1, RXRA, STAT

^o^Dnd41 TCellLeukemia cell line

^p^Foxp1, HDAC2, Hoxa10, Hoxa9, Hoxd10

### The risk allele of the intergenic 6q23 variant rs6927172 correlates with increased expression of *IL20RA*

We next focused on confirming disease causal genes by exploring the effect of SNP genotype on gene expression levels. However, publicly available eQTL data from different human tissues, including B-lymphoblastoid cell lines (LCLs), revealed no cis-eQTLs with the disease-associated SNPs (rs6920220, rs7752903 and rs610604) or SNPs in LD (r^2^ > 0.8) with them.

**Fig. 3:**
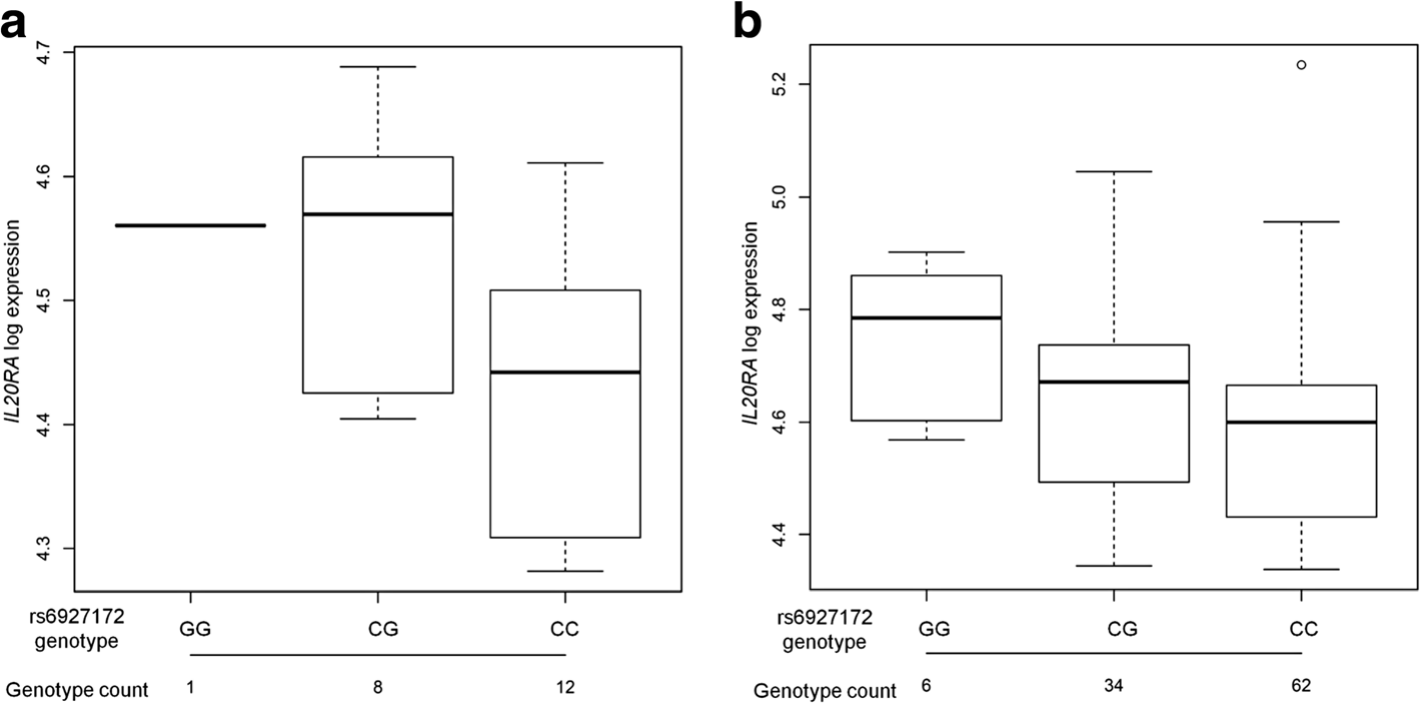
eQTL effect of rs6927172 on gene expression. **a** Increased expression of *IL20RA* in primary CD4+ T-cells from 21 healthy individuals carrying the G risk allele of rs6927172, *P* = 0.02. **b** Increased expression of *IL20RA* in primary CD4+ T-cells from 102 early inflammatory arthritis clinic patients carrying the risk G allele of rs6927172, *P* = 0.03. The three different genotypes for the SNPs are displayed on the *x-axis* and gene expression levels on the *y-axis. Error bars* indicate standard deviation

Since gene expression is cell type specific, the effect of SNPs on transcription may occur in disease-relevant cell types only. To study the correlation between 6q23 SNP genotypes and gene expression levels in autoimmune relevant cell types, whole genome expression data from CD4+ and CD8+ primary T-cells obtained from 21 individuals from the Arthritis Research UK National Repository of Healthy Volunteers (NRHV) were interrogated. In CD4+ T-cells, the risk allele of rs6927172 correlated with increased expression of the *IL20RA* gene (Fig. 3a, *P* = 0.02), supporting that the physical interaction between them plays a functional role in the transcriptional control of *IL20RA* (Fig. 1). Additionally, CD4+ T-cell whole genome expression data were available from a cohort of 102 early undifferentiated arthritis patients collected at baseline. To avoid confounding by clinical epiphenomena typically seen in patients, individuals that were diagnosed with RA after follow-up were not included in the analysis. The correlation between rs6927172 risk alleles and increased expression of *IL20RA* was validated in this larger cohort (Fig. 3b, *P* = 0.03). No correlation was found between disease-associated SNPs (rs6927172, rs7752903 or rs610604) and expression of the previously assumed target, *TNFAIP3*, or the other interacting genes, including *IFNGR1*, in any of the CD4+ or CD8+ T-cell cohorts. Whole genome expression data were also available in primary CD19+ B-cells for the same cohort, but no eQTLs were detected for rs6927172, rs7752903 or rs610604, suggesting that the effect of rs6927172 on *IL20RA* expression may either be T-cell type specific or stimulation-dependent in B-cells. Therefore, the eQTL results showing that 6q23 non-coding variants are correlated with *IL20RA* messenger RNA (mRNA) expression in CD4+ T-cells further support that *IL20RA* is one of the target genes in the region, as evidenced by the CHi-C experiment.

### rs6927172 risk allele shows higher frequency of interactions with *IL20RA* and *IFNGR1*

**Fig. 4:**
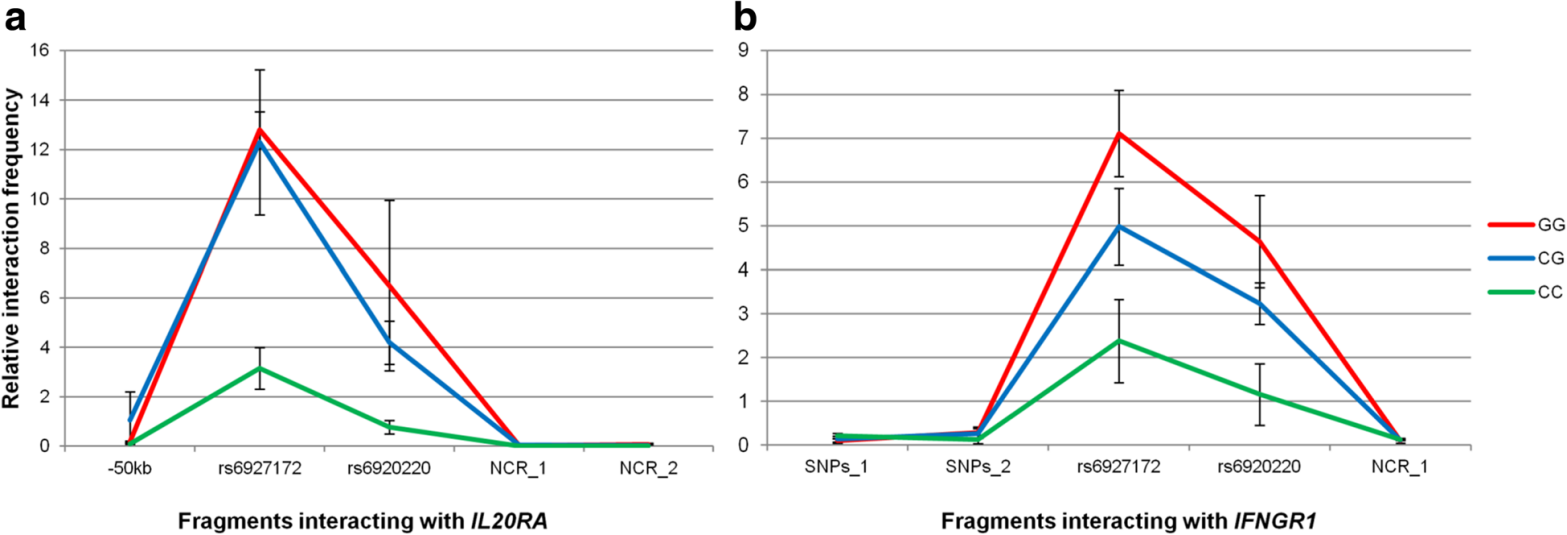
Genotype-specific 3C showing preferential interaction of the disease risk G allele of rs6927172 with *IL20RA* (**a**) and *IFNGR1* (**b**). *–50 kb* restriction fragment located 50 kb upstream of the rs6927172 containing restriction fragment, *rs6927172* restriction fragment containing rs6927172, *rs6920220* restriction fragment containing the top GWAS SNP in the 6q23 region, *NCR* non-interacting control region. *Error bars* indicate standard deviation of three biological replicates

Having established that the non-coding 6q23 SNPs interact with several genes by long-range chromatin looping, we investigated whether the different alleles of rs6927172, the most likely candidate regulatory SNP according to bioinformatic analysis, interact with different affinities with their targets. Evaluation of 3C interactions was carried out in LCLs, as they have been genotypically well characterised as part of the HapMap Project and cells carrying the three different genotypes for the rs6927172 variant (GM11993 CC, GM12878 CG and GM07037 GG) are readily accessible commercially. This experiment revealed significantly higher interaction frequencies between both *IL20RA* and *IFNGR1* and the restriction fragment containing rs6927172 in individuals carrying the risk G allele of this SNP compared with the homozygous non-risk allele (GG versus CC, *P* = 0.01; CG versus CC, *P* = 0.01 and GG versus CC, *P* = 0.04; CG versus CC, *P* = 0.02, respectively) (Fig. 4). Interaction frequencies between the fragment containing rs6927172 and both fragments containing the lncRNAs RP11-10J5.1 and RP11-240M16.1 were similar regardless of genotype (Additional file 1: Figure S2). Similarly, none of the interactions between *TNFAIP3* and targets identified in the CHi-C experiment (*PTPN11* pseudogene, RP11-10J5.1, RP11-240M16.1, Y_RNA and *IL20RA*) and between *IL20RA* and RP11-10J5.1 were influenced by rs6927172 genotype (Additional file 1: Figure S3).

6q23 is one of the most important loci for RA susceptibility, being the third most strongly associated region after *HLA*-*DRB1* and *PTPN22*. Although T-cells are thought to be the most important cell type in RA pathogenesis, synovial fibroblasts have also been shown to play a crucial role in the perpetuation of disease [35]. Therefore, we sought to evaluate the 3D conformation of the locus in this cell type. The preferential interaction of the fragment containing rs6927172 and *IL20RA* was confirmed by 3C-qPCR in primary human synovial fibroblasts (Additional file 1: Figure S4).

Hence, our experiments suggest that increased *IL20RA* expression that correlates with the risk G allele of rs6927172 may be mediated through increased ability to bind the *IL20RA* gene via chromatin looping.

### The risk allele of rs6927172 shows increased enrichment of regulatory proteins

**Fig. 5:**
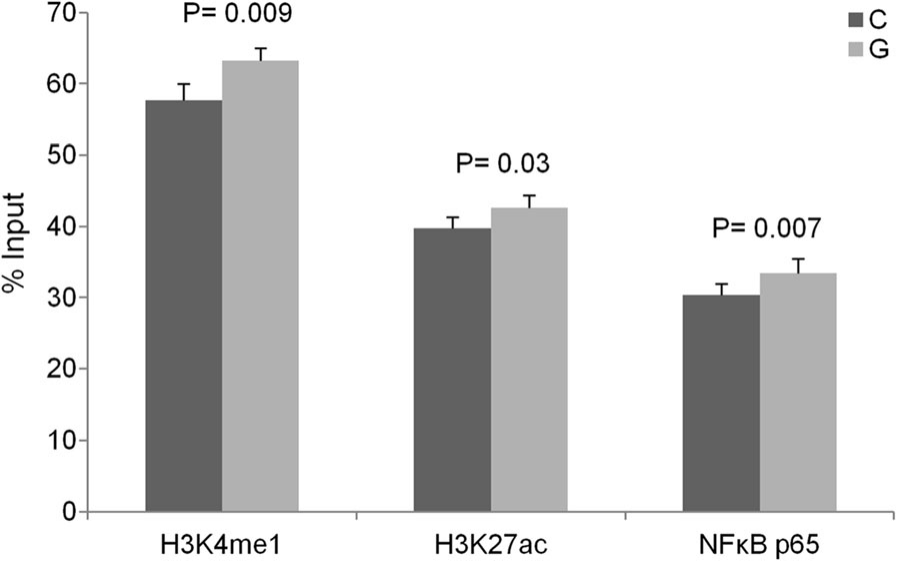
Allele-specific ChIP in Jurkat cells, showing increased binding of H3K4me1, H3K27ac and NFκB p65 to the risk allele of rs6927172. *Error bars* indicate standard deviation of three biological replicates

To further explore the role of rs6927172 in transcriptional regulation, we evaluated enrichment of chromatin marks of active regulatory elements to this site using chromatin immunoprecipitation (ChIP) in LCLs. We observed an enrichment of histone marks, H3K4me1 and H3K27ac, to the region containing the SNP, compared to a non-regulatory control region (*P* = 0.0001 and *P* = 0.0001, respectively) and to a no antibody control sample (*P* = 0.0001 and *P* = 0.0008, respectively), confirming the bioinformatic evidence that rs6927172 is located in a regulatory element (Additional file 1: Figure S5). We then performed allele-specific qPCR using Taqman probes complementary to each rs6927172 allele in Jurkat T-cells and GM12145 B-cells, which are both heterozygous for the variant, and the balance between the immunoprecipitated fragments with the C allele or the G allele was determined. In Jurkat cells, the risk G allele showed evidence of increased enrichment of both H3K4me1 (*P* = 0.009) and H3K27ac (*P* = 0.03), compared to the non-risk allele (Fig. 5), supporting the CD4+ specific nature of the eQTL evidence and further suggesting that the risk allele is correlated with an increased regulatory activity. By contrast, in GM12145 B-cells, where no eQTL evidence was detected/observed, the non-risk C allele showed evidence of increased enrichment for histone marks (*P* = 0.009 and *P* = 0.0001 for H3K4me1 and H3K27ac respectively), further highlighting the cell type specificity of transcriptional regulation (Additional file 1: Figure S5).

The rs6927172 variant was predicted to alter the binding motif for eight transcription factors, including NFκB and BCL3 (Table 1). Since NFκB is an important mediator of the immune response [36] and previous studies have shown that the TT > A variant, which maps to the *TNFAIP3* LD block tagged by rs7752903, impairs the binding of this transcription factor [9], we experimentally tested whether NFκB binds rs6927172 alleles with different affinities. We performed ChIP in Jurkat and GM12878 cell lines using antibodies for the p50 and p65 subunits of NFκB. Estimation of the C/G ratio in the immunoprecipitated chromatin was performed and results showed that, in Jurkat cells, the p65 subunit of NFκB binds with higher affinity to the risk G allele, compared to the non-risk C allele (*P* = 0.007) (Fig. 5). The SNP did not show evidence of altered binding of NFκB in the B-lymphoblastoid cell line.

BCL3 is a transcriptional co-activator that inhibits the nuclear translocation of the NFκB p50 subunit in the cytoplasm and contributes to the regulation of transcription of NF-κB target genes in the nucleus [37–39]. Therefore, we also investigated binding of BCL3 to the different alleles of rs6927172 using the same approach. Although this transcription factor seems to be part of the transcriptional machinery at the site of the SNP, BCL3 binding showed no statistically significant differences between the two alleles, either in Jurkat or in GM12878 cells.

Taken together, these results suggest that the mechanism by which the risk allele of rs6927172 increases expression of *IL20RA* may be mediated by an increased regulatory activity and augmented binding of the transcription factor NFκB.

## Discussion

The chromosomal region 6q23 is an important locus in autoimmunity. It is an exemplar complex non-coding genomic region, some distance from the nearest gene, containing enhancer elements and implicated in multiple diseases by GWAS, but where independent variants associate with different conditions. To date, investigation of the functional consequences of disease-associated alleles have focused almost exclusively on the gene *TNFAIP3*. Here we present findings from a hypothesis-free, systematic approach using the recently developed CHi-C method to identify causal genes at this locus. Our experiments have revealed that the spatial organisation of the chromatin at this region is complex, bringing together several genes with key roles in the immune response, including *IL20RA*, *IFNGR1* and *TNFAIP3*, alongside regulatory elements containing SNPs associated with different autoimmune diseases. This supports the recently proposed concept of specialised transcription factories, where co-regulated genes come together to share transcription factors and regulatory elements such as enhancers [40].

Previous studies investigating the functional role of 6q23 disease variants had been restricted to the SNPs mapping to the LD block tagged by rs7752903 spanning the *TNFAIP3* gene, associated with SLE, RA and celiac disease, showing that the TT > A variant, located downstream of *TNFAIP3*, impairs that gene’s expression through chromatin looping and altered NFκB binding [9, 30, 31, 40]. However, the functional impact of the remaining disease-associated SNPs at the locus, such as the intergenic rs6920220 nominally assigned to *TNFAIP3*, had remained unexplored. Our CHi-C study, supplemented by confirmatory 3C, eQTL and ChIP evidence, offers for the first time a firm indication that autoimmune-associated regions in general [21], and this region in particular, can demonstrate complex regulatory interactions with a number of plausible candidate genes, potentially functional lncRNA genes and, importantly, each other. The complexity of the interactions is magnified when considering the differences observed in cell types (here, in B and T-cell lines and synovial fibroblasts). Interestingly, the rs6927172 alleles, associated with RA, correlate with *IL20RA* expression levels in CD4+ T-cells, supporting the accumulating evidence that CD4+ T-cells are the most relevant cell type to RA [41]. Published high resolution Hi-C data were available for GM12878 B-lymphoblastoid cells and we observed numerous, strong interactions between the 6q23 intergenic SNPs and *IL20RA*, supporting our results [42]. In contrast, these interactions with the associated intergenic region were markedly decreased or non-existent in cell lines that do not express *IL20RA*, such as human umbilical vein endothelial cells (HUVEC) or chronic myeloid leukaemia (K562) cells (Additional file 1: Figure S7), supporting a cell type dependent regulatory role for the disease-associated enhancer region and *IL20RA*.

Chromatin looping and eQTL experiments strongly support *IL20RA* as a putative causal autoimmunity gene in 6q23. The *IL20RA* gene encodes the IL-20 receptor α subunit (IL-20RA), which can form a heterodimeric receptor with either IL-20RB to bind IL-19, IL-20 and IL-24, or with IL-10RB to bind IL-26 [43]. Evidence suggests that this family of cytokines have a pro-inflammatory effect, and are essential in the activation of the epithelial innate immunity [44], with expression of *IL20RA* detected in whole blood, T-cells, B-cells and monocytes [45]. Recently, interactions of IL-20 subfamily cytokines with their receptors have been shown to be involved in the pathogenesis of RA. IL-20 and its receptors are upregulated in the synovium of RA patients [46–50] and IL-19, IL-20 and IL-22 are able to increase the proliferation of synovial cells and induce IL-6, IL-8 and CCL2 in these cells [48, 50]. In rats, experimentally induced autoimmune arthritis and collagen-induced arthritis are attenuated by IL-19 blockade [51] and administration of soluble IL-20RA [47, 51], respectively. These cytokines also have involvement in skin inflammation [52]. Overexpression of *Il20*, *Il22* or *Il24* in mice leads to the development of psoriasis-like skin lesions [53–55], and levels of IL-19, IL-20, IL-22 and IL-24 are increased in psoriatic skin [56–58]. Notably, SNPs mapping to the *TNFAIP3* region have been shown to be associated with Ps and PsA, but map to a different risk haplotype, tagged by rs610604, distinct to other autoimmune diseases [22, 26]. Very interestingly, two recent clinical trials have demonstrated that anti-IL-20 monoclonal antibody is effective in the treatment of RA and psoriasis [59, 60]. Furthermore, levels of IL-19, IL-20, IL-24 and IL-26 are also elevated in serum of patients with inflammatory bowel disease [61–64], which is associated with the intergenic 6q23 variants tagged by rs6920220 [25]. The evidence that SNPs associated with different autoimmune diseases interact with each other and the same genes supports a concept that regional genetic variation, regulating similar target genes, but with mechanistic and cellular differences, are risk factors for different diseases. This may also suggest that blocking the IL-20 pathway might be effective in the treatment of multiple autoimmune diseases. Indeed, a recent study has shown that selecting a therapeutic target with genetic data supporting its role could double the chance of a drug’s success in clinical improvement [65].

Our CHi-C experiment suggested another potential novel causal gene in the 6q23 region, *IFNGR1*. In addition, targeted 3C experiments found that the interaction between rs6927172 and this gene is stronger when the disease risk G allele is present. *IFNGR1* encodes one of the subunits of the interferon gamma (IFN-γ) receptor. This cytokine plays an important role in autoimmunity, since it is involved in macrophage activation, enhanced MHC expression on neighbouring cells, balancing Th1/Th2 cell differentiation, and inducing the secretion of other pro-inflammatory cytokines [66]. Although it has been shown that an increased expression of *IFNGR1* in blood is associated with RA [67], we did not detect an effect of rs6927172 genotype on this gene’s expression levels in CD4+ and CD8+ T cells. eQTLs, though, are context-specific [6, 68–72] and, therefore, it would be interesting to explore whether the SNP influences *IFNGR1* expression in other cell types and/or under different stimulatory conditions.

Whereas we provide evidence of additional putative causal genes in the 6q23 region, the *TNFAIP3* gene remains a strong candidate. The role of *TNFAIP3* in autoimmunity is well established. The protein encoded by *TNFAIP3*, A20, is induced by tumour necrosis factor (TNF) and inhibits NFκB activation and TNF-mediated apoptosis [73]. Mice deficient for A20 develop severe multiorgan inflammation [74] and tissue-specific deletion of A20 results in different phenotypes that resembles human autoimmune diseases such as inflammatory polyarthritis (macrophages), SLE (dendritic cells), IBD (intestinal epithelial cells) or psoriasis (keratinocyes) [73].

Bioinformatic analysis suggested that rs6927172 is the most likely causal SNP in the rs6920220 LD block. Genotype specific 3C showed increased interactions with the *IL20RA* gene when the risk G allele is present compared with the non-risk allele. By contrast, the genotype-specific interaction was not observed for the rs6920220 variant. However, although bioinformatic evidence and ChIP experiments coupled with previous evidence from electrophoretic mobility shift assays [34] point to rs6927172 as the most likely causal SNP, the resolution of this experiment is limited by the restriction enzyme used; rs6927172 is located in the same restriction fragment as rs35926684 and both SNPs are strongly correlated (r^2^ = 0.8). Therefore, although bioinformatic evidence suggests that rs35926684 is less likely to affect binding of regulatory proteins, the possibility that it is the causal SNP, or that both SNPs contribute to transcriptional regulation, cannot be excluded.

Our study illustrates the challenges in linking associated variants to function. Associated variants can be linked to a number of genes, dependent on which enhancer they are located within and the cell type under investigation. This could explain apparent inconsistencies in findings; for example, how the risk variant of rs6927172 is associated with higher levels of active enhancer histone marks in Jurkat cells, but has the opposite effect in GM12878 cells. Indeed, up to 50 % of allele specific associations with epigenetic marks of enhancer activity (histoneQTLs) have been reported to show inconsistent direction of effects between samples, indicating the intricacies that exist in gene regulation [75]. Nonetheless, our work reinforces previous evidence that the nearest plausible biological candidate gene is not necessarily the causal gene. While *TNFAIP3* gene involvement is still implicated at the 6q23 locus, the primary causal gene may well be *IL20RA*, supported by the success of anti-IL20 therapies in RA and Ps.

It is noteworthy that the intergenic 6q23 SNP, correlated with higher frequency of interactions with *IL20RA*, higher expression of *IL20RA* and increased enrichment of histone marks of active enhancers and NFκB, is located at the boundary of two topologically associated domains (TADs) (Fig. 1g and j). TADs are genomic regions that show high levels of interaction within the region and little or no interaction with bordering regions and are thought to be conserved across different cell types and species [76, 77]. It has been shown that boundaries between TADs can separate functionally distinct regions of the genome [78]. Intriguingly, it has been suggested that eQTLs often occur around TAD boundaries and preferentially associate with genes across domains [79]. There is now evidence that disruption of TAD boundaries can cause ectopic interactions between regulatory non-coding DNA and gene promoters, resulting in pathogenic phenotypes [80]. Our CHi-C experiments show long-range interactions between *IL20RA* and targets located outside the TAD this gene is located, i.e. the intergenic disease-associated SNPs, *TNFAIP3* and several lncRNAs (Fig. 1). The cell lines used in these experiments (GM12878 and Jurkat) are both heterozygous for rs6927172 and genotype-specific 3C experiments showed that the interaction between this SNP and *IL20RA* occurs preferentially when the risk allele is present (Fig. 3). It would be interesting to explore whether this autoimmunity associated variant exerts its pathogenic effect through a disruption of the TAD boundary between *IL20RA* and potential regulatory elements that would not otherwise interact with it.

## Conclusions

We provide evidence that an intergenic enhancer region on 6q23, associated with numerous autoimmune diseases and nominally assigned to *TNFAIP3* although over 200 kb from the nearest gene, makes allele-specific, regulatory contact with *IL20RA*, the target of an existing drug and located 680 kb away from the associated region. Our findings show how functional evaluation of disease risk loci can help better translate GWAS findings into biologically meaningful mechanisms of disease and validate existing therapeutic targets or suggest potential new ones.

## Methods

### Cell culture

B-lymphoblastoid cell lines (LCL) were obtained from the Coriell Institute for Medical Research (Additional file 1: Table S2). Cells were grown in vented 25 cm^2^ cell culture flasks containing 10–20 mL of Roswell Park Memorial Institute medium (RPMI)-1640 + 2 mM L-glutamine culture medium, supplemented with 15 % fetal bovine serum (FBS). Flasks were incubated upright at 37 °C/5 % CO_2_. Cultures were regularly monitored to maintain a cell density in the range of 2 × 10^5^–5 × 10^5^viable cells/mL. Cells were split every two days into fresh medium until they reached a maximum density of 1 × 10^6^ cells/mL.

Jurkat E6.1 human leukaemic T-lymphoblast cells were obtained from LGC Standards. Cells were grown in vented 25 cm^2^ cell culture flasks containing 10–20 mL of RPMI-1640 + 2 mM L-glutamine, supplemented with 10 % FBS. Flasks were incubated upright at 37 °C/5 % CO_2_ and the cultures regularly monitored to maintain a cell density in the range of 3 × 10^5^–9 × 10^5^ viable cells/mL.

These cell lines are not listed in the in the database of commonly misidentified cell lines maintained by ICLAC, were authenticated using STR analysis and were tested for mycoplasma contamination (MycoSEQ® Mycoplasma Detection System, 4460625, Life Technologies).

### Capture Hi-C

Chromatin interactions at 6q23 were scrutinised using CHi-C as part of a larger study that included all confirmed risk loci for four autoimmune diseases (RA, JIA, PsA and T1D) [21].

We tested chromatin interactions in two complementary experiments: Region Capture, which targeted regions associated with disease [22, 27, 81–83], and Promoter Capture, which provided independent validation by capturing all gene promoters within 500 kb upstream and downstream of lead disease-associated SNPs. Associated regions were defined by selecting all SNPs in LD with the lead disease-associated SNP (r^2^ ≥ 0.8; 1000 Genomes phase 1 EUR samples; May 2011). For the Promoter Capture experiment, HindIII restriction fragments were identified within 500 bp of the transcription start site of all genes mapping to the defined regions (*Ensembl *release 75; GRCh37). A control region with well characterised long-range interactions was also included, *HBA* [84]. Capture oligos (120 bp; 25–65 % GC, <3 unknown (N) bases) were designed using a custom Perl script within 400 bp but as close as possible to each end of the targeted HindIII restriction fragments.

Human T-cell (Jurkat) and B-cell (GM12878) lines were used, since they are among the most relevant cell types in autoimmune disease [5]. Hi-C libraries were generated as previously described [85]. Cells of 5–6 × 10^7^ were grown to ~90 % confluence and cross-linked with 2 % formaldehyde for 10 min at room temperature. The cross-linking reaction was quenched by adding cold 1 M glycine to a final concentration of 0.125 M for 5 min at room temperature, followed by 15 min on ice. Cells were resuspended in 50 mL ice-cold lysis buffer (10 mM Tris–HCl pH 8, 10 mM NaCl, 0.2 % Igepal CA-630, protease inhibitors) and lysed on ice for 30 min, with 2 × 10 strokes of a Dounce homogeniser. Following lysis, the nuclei were pelleted and washed with 1.25 × NEB Buffer 2 then resuspended in 1.25 × NEB Buffer 2. Hi-C libraries were digested using HindIII then prepared as described in van Berkum et al. [86] with modifications described in Dryden et al. [18]. Pre-Capture amplification was performed with eight cycles of PCR on multiple parallel reactions from Hi-C libraries immobilised on Streptavidin beads which were pooled post-PCR and SPRI bead purified. The final library was resuspended in 30 μL TLE (10 mM Tris pH8; 0.1 mM EDTA) and the quality and quantity assessed by Bioanalyzer and qPCR.

Hybridisation of Agilent SureSelect custom Promoter and Region Capture RNA bait libraries to Hi-C libraries was carried out using Agilent SureSelectXT reagents and protocols. Post-capture amplification was carried out using six cycles of PCR from streptavidin beads in multiple parallel reactions, then pooled and purified using SPRI beads.

Two biological replicates for each of the cell lines were prepared for each target capture. Sequencing was performed on Illumina HiSeq 2500 generating 75 bp paired-end reads (Genomic Technologies Core Facility in the Faculty of Life Sciences, University of Manchester). CASAVA software (v1.8.2, Illumina) was used to make base calls; reads failing Illumina filters were removed before further analysis. Promoter Capture libraries were each sequenced on one HiSeq lane and each Region Capture library was sequenced on 0.5 of a HiSeq lane. Sequences were output in FASTQ format, poor quality reads truncated or removed as necessary, using Trimmomatic version 0.30 [87], and subsequently mapped to the human reference genome (GRCh37/hg19) and filtered to remove experimental artefacts using the Hi-C User Pipeline (HiCUP, http://www.bioinformatics.babraham.ac.uk/projects/hicup/). Off-target di-tags, where neither end mapped to a targeted fragment, were removed from the final datasets.

Di-tags separated by <20 kb were removed prior to analysis, as 3C data have shown a very high interaction frequency within this distance [88]. Significant interactions for cis interactions within 5 Mb were determined using the ‘High resolution analysis of cis interaction peaks’ method described by Dryden et al. [18]. To correct for experimental biases, the interactability of each fragment was calculated to long-range, ‘*trans*’ fragments, under the assumption that those represent random, background interactions and so should be similar in any particular baited fragment. The resulting distribution is bimodal consisting of stochastic noise (low *trans* counts) and genuine signal (high *trans* counts). A truncated negative binomial distribution was fitted to the distribution. The 5 % quantile point of the non-truncated distribution was determined to provide the noise threshold. A negative binomial regression model was fitted to the filtered data correcting for the interactability of the captured restriction fragment and interaction distance. For interactions where both the target and baited region were captured (double-baited interactions) we also accounted for the interactability of the other end.

Interactions were considered statistically significant after combining replicates and filtering on FDR ≤ 5 %. Significant interactions were visualised in the WashU Epigenome Browser [89, 90].

### Chromosome conformation capture (3C)

Validation of interactions was carried out on biological replicate 3C libraries for each of the cell lines (GM12878 and Jurkat). Libraries were prepared using the cross-linking, digestion with HindIII and ligation steps used for the generation of Hi-C libraries [84] but without the biotin fill-in step. qPCR was carried out using Power SYBR® Master Mix (Life Technologies) according to the manufacturer’s instructions using the following cycling conditions: 50 °C 2 min, 95 °C 10 min, followed by 40 cycles of 95 °C 15 s, 60 °C 1 min. qPCR was performed in triplicate using 50 ng of 3C library [88]. Standard curves for each primer set used in the qPCR were generated using tenfold serial dilutions of 3C control template libraries, prepared by digestion and random ligation of bacterial artificial chromosomes (BACs) (Life Technologies) spanning the region of interest with minimal overlap (Additional file 1: Table S3). Data were normalised to a short-range ligation product using the bait primer in combination with a primer for adjacent HindIII fragments, to control for differences in cross-linking and ligation efficiencies between different cell lines. 3C primers are shown in Additional file 1: Table S4. Statistical analysis was performed in STATA by paired t-test. *P* values < 0.05 were considered statistically significant. Variance between groups was similar (two-tailed F-test for equality of two variances *P* > 0.05).

### Bioinformatics

To narrow down the most plausible causal SNP among all variants in LD with the lead GWAS SNP rs6920220, Haploreg v4.1 was used with the following settings: LD threshold, r^2^ ≥ 0.8; 1000G Phase 1 population for LD calculation: EUR; Source for epigenomes: ChromHMM (25-state model using 12 imputed marks); Mammalian conservation algorithm: SiPhy-omega. Additionally, RegulomeDBVersion 1.1 was used.

### Expression quantitative trait loci (eQTLs) analysis

Public eQTL data were interrogated using Haploreg v4.1 [32], which examines all datasets obtained from the GTEx analysis release V6 (http://www.gtexportal.org/static/datasets/gtex_analysis_v6/single_tissue_eqtl_data/GTEx_Analysis_V6_eQTLs.tar.gz), the GEUVADIS analysis (EUR and YRI panels, http://www.ebi.ac.uk/arrayexpress/files/E-GEUV-1/analysis_results/), the NCBI eQTL Browser (http://www.ncbi.nlm.nih.gov/projects/gap/eqtl/index.cgi, lymphoblastoid cell lines [91, 92], liver [93] and brain [94]) and eight additional studies including data obtained from tumours [95], blood [96], lung [97], heart [98], monocytes [4], bone [99], lymphoblastoid cell lines [100] and brain [101].

Four whole genome gene expression datasets were available: CD4+ and CD8+ T-cells from 21 healthy individuals of the National Repository of Healthy Volunteers (NRHV), The University of Manchester (North West Centre for Research Ethics Committee) (Additional files 2, 3, 4 and 5), and CD4+ T-cells and CD19+ B-cells from 102 early undifferentiated arthritis patients, Newcastle University (Newcastle and North Tyneside Local Research Ethics Committee) (Additional files 6, 7, 8 and 9). Informed consent was obtained from all participants. mRNA was isolated from sorted cell subsets, quality and concentration assessed using the Agilent Bioanalyzer and Nanodrop, before complementary DNA (cDNA)/complementary RNA (cRNA) conversion using Illumina TotalPrep RNA Amplification Kits. A total of 750 ng of cRNA was hybridised to HumanHT-12 v4 Expression BeadChip arrays according to the manufacturer’s protocol before being scanned on the Illumina iScan system. Raw expression data were exported from Illumina GenomeStudio and analysed using the R Bioconductor package ‘limma’ [102]. Briefly, the neqc function was used for log2 transformation of the data, background correction and quantile normalisation using control probes. Principal component analysis was used to detect batch effects. The cDNA/cRNA conversion produced the largest batch effect in both cohorts and was corrected using ComBat (in R Bioconductor package sva) (http://bioconductor.org/packages/release/bioc/html/sva.html). Genome-wide genotype data were generated using the Illumina HumanCoreExomeBeadChip kit. Genotype data were aligned to the 1000 genomes reference strand, pre-phased using SHAPEIT2 (v2.r727 or v2.r790), before imputation using IMPUTE2 (v2.3.0 or v2.3.1) with the 1000 genome reference panel (Phase 1, December 2013 or June 2014). Imputed data were hard-called to genotypes using an INFO score cutoff of 0.8 and posterior probability of 0.9. The effect of the SNPs on gene expression was analysed using MatrixEQTL (v.2.1.0) (http://www.bios.unc.edu/research/genomic_software/Matrix_eQTL/) with an additive linear model. The errorCovariance = numeric() parameter was set to account for possible differences in variance between groups. SNPs within 4 Mb of a gene expression probe were considered to be cis-eQTL, since the majority (99 %) of interactions detected in the CHi-C experiment happened within a 4 Mb window. *P* values < 0.05 were considered statistically significant. The study (N = 102 early arthritis patients) had 80 % power to detect a change of 0.08 log expression at 5 % significance level.

### Chromatin immunoprecipitation (ChIP)

1 × 10^7^ cells were cross-linked with 1 % formaldehyde for 10 min at room temperature. Cells were lysed in 1 mL of ChIP lysis buffer (50 mM Tris–HCl pH8.1, 10 mM EDTA, 1 % SDS, one protease inhibitor cocktail tablet) and chromatin sheared using a Covaris S220 with the following conditions: target base pairs: 200–400 bp, duty cycle: 5 % for LCL; 10 % for Jurkat cells, peak incident power: 140 Watts, cycles per burst: 200, temperature: 4 °C, time: 20–25 min.

Each immunoprecipitation (IP) was carried out in triplicate using LCLs obtained from HapMap individuals (Additional file 1: Table S1). The negative control was a no antibody control or IgG. Antibodies were available from Abcam for NFκB p50 (ab7971), NFκBp65 (ab7970), H3K4me1 (ab8895) and H3K27ac (ab4729) and from Santa Cruz for BCL3 (sc-185X). To detect the relative enrichment of regions interacting with the target protein, qPCR of ChIP and input samples was carried out. qPCR was performed in triplicate using SYBR green, or TaqMan probes complementary to each allele of rs6927172 for allele-specific assays (Applied Biosystems, assay ID C___1575580_100), on an Applied Biosystems QuantStudio 12 K Flex qPCR instrument. Primers were designed for the target SNP region, positive control region and negative control region (Additional file 1: Table S5). Following qPCR, the % input for each sample was calculated and statistical analysis of ChIP data was carried out to determine significant differences in antibody binding to the different SNP genotypes in STATA by paired t-test. *P* values < 0.05 were considered statistically significant. Variance between groups was similar (two-tailed F-test for equality of two variances *P* > 0.05).

## Additional files

Additional file 1: Supplementary tables and figures. (DOCX 2089 kb)
Additional file 2: Contains the cis-eQTLs with *P* value < 5 % for CD4+ T-cells obtained from NRHV participants. (TXT 405 kb)
Additional file 3: Contains the normalised expression values for CD4+ T-cells obtained from NRHV participants. (TXT 26 kb)
Additional file 4: Contains the cis-eQTLs with *P* value < 5 % for CD8+ T-cells obtained from NRHV participants. (TXT 352 kb)
Additional file 5: Contains the normalised expression values for CD8+ T-cells obtained from NRHV participants. (TXT 26 kb)
Additional file 6: Contains the cis-eQTLs with *P* value < 5 % for B-cells obtained from early undifferentiated arthritis patients. (TXT 985 kb)
Additional file 7: Contains the normalised expression values for B-cells obtained from early undifferentiated arthritis patients. (TXT 124 kb)
Additional file 8: Contains the cis-eQTLs with *P* value < 5 % for T-cells obtained from early undifferentiated arthritis patients. (TXT 1162 kb)
Additional file 9: Contains the normalised expression values for T-cells obtained from early undifferentiated arthritis patients. (TXT 125 kb)

## Abbreviations

3C: Chromosome conformation capture
BACs: Bacterial artificial chromosomes
CeD: Celiac disease
CHi-C: Capture Hi-C
CHiCAGO: Capture HiC Analysis of Genomic Organisation
ChIP: Chromatin immunoprecipitation
eQTL: Quantitative trait loci
FBS: Fetal bovine serum
GWAS: Genome-wide association studies
HiCUP: Hi-C User Pipeline
IBD: Inflammatory bowel disease
IFN-γ: Interferon gamma
IL-20RA: IL-20 receptor α subunit
LCLs: B-lymphoblastoid cell lines
LD: Linkage disequilibrium
lncRNAs: Long non-coding RNAs
NRHV: National Repository of Healthy Volunteers
Ps: Psoriasis
PsA: Psoriatic arthritis
qPCR: Quantitative real-time PCR
RA: Rheumatoid arthritis
RPMI: Roswell Park Memorial Institute medium
SLE: Systemic lupus erythematosus
SNPs: Single nucleotide polymorphisms
T1D: Type 1 diabetes
TADs: Topologically associated domains
*TNFAIP3*: Tumour necrosis factor alpha-induced protein 3

## References

[1] Welter D, MacArthur J, Morales J, Burdett T, Hall P, Junkins H (2014). The NHGRI GWAS Catalog, a curated resource of SNP-trait associations. Nucleic Acids Res.

[2] Freedman ML, Monteiro AN, Gayther SA, Coetzee GA, Risch A, Plass C (2011). Principles for the post-GWAS functional characterization of cancer risk loci. Nat Genet.

[3] Ward LD, Kellis M (2012). Interpreting noncoding genetic variation in complex traits and human disease. Nat Biotechnol.

[4] Fairfax BP, Humburg P, Makino S, Naranbhai V, Wong D, Lau E (2014). Innate immune activity conditions the effect of regulatory variants upon monocyte gene expression. Science.

[5] Farh KK, Marson A, Zhu J, Kleinewietfeld M, Housley WJ, Beik S (2015). Genetic and epigenetic fine mapping of causal autoimmune disease variants. Nature.

[6] Ye CJ, Feng T, Kwon HK, Raj T, Wilson MT, Asinovski N (2014). Intersection of population variation and autoimmunity genetics in human T cell activation. Science.

[7] Davison LJ, Wallace C, Cooper JD, Cope NF, Wilson NK, Smyth DJ (2012). Long-range DNA looping and gene expression analyses identify DEXI as an autoimmune disease candidate gene. Hum Mol Genet.

[8] Pomerantz MM, Ahmadiyeh N, Jia L, Herman P, Verzi MP, Doddapaneni H (2009). The 8q24 cancer risk variant rs6983267 shows long-range interaction with MYC in colorectal cancer. Nat Genet.

[9] Wang S, Wen F, Wiley GB, Kinter MT, Gaffney PM (2013). An enhancer element harboring variants associated with systemic lupus erythematosus engages the TNFAIP3 promoter to influence A20 expression. PLoS Genet.

[10] Zhang X, Cowper-Sal IR, Bailey SD, Moore JH, Lupien M (2012). Integrative functional genomics identifies an enhancer looping to the SOX9 gene disrupted by the 17q24.3 prostate cancer risk locus. Genome Res.

[11] Bulger M, Groudine M (2011). Functional and mechanistic diversity of distal transcription enhancers. Cell.

[12] Sanyal A, Lajoie BR, Jain G, Dekker J (2012). The long-range interaction landscape of gene promoters. Nature.

[13] Schoenfelder S, Furlan-Magaril M, Mifsud B, Tavares-Cadete F, Sugar R, Javierre BM (2015). The pluripotent regulatory circuitry connecting promoters to their long-range interacting elements. Genome Res.

[14] Dekker J, Rippe K, Dekker M, Kleckner N (2002). Capturing chromosome conformation. Science.

[15] Dostie J, Richmond TA, Arnaout RA, Selzer RR, Lee WL, Honan TA (2006). Chromosome Conformation Capture Carbon Copy (5C): a massively parallel solution for mapping interactions between genomic elements. Genome Res.

[16] Simonis M, Klous P, Splinter E, Moshkin Y, Willemsen R, de WE (2006). Nuclear organization of active and inactive chromatin domains uncovered by chromosome conformation capture-on-chip (4C). Nat Genet.

[17] Lieberman-Aiden E, van Berkum NL, Williams L, Imakaev M, Ragoczy T, Telling A (2009). Comprehensive mapping of long-range interactions reveals folding principles of the human genome. Science.

[18] Dryden NH, Broome LR, Dudbridge F, Johnson N, Orr N, Schoenfelder S (2014). Unbiased analysis of potential targets of breast cancer susceptibility loci by Capture Hi-C. Genome Res.

[19] Jager R, Migliorini G, Henrion M, Kandaswamy R, Speedy HE, Heindl A (2015). Capture Hi-C identifies the chromatin interactome of colorectal cancer risk loci. Nat Commun.

[20] Mifsud B, Tavares-Cadete F, Young AN, Sugar R, Schoenfelder S, Ferreira L (2015). Mapping long-range promoter contacts in human cells with high-resolution capture Hi-C. Nat Genet.

[21] Martin P, McGovern A, Orozco G, Duffus K, Yarwood A, Schoenfelder S (2015). Capture Hi-C reveals novel candidate genes and complex long-range interactions with related autoimmune risk loci. Nat Commun.

[22] Bowes J, Budu-Aggrey A, Huffmeier U, Uebe S, Steel K, Hebert HL (2015). Dense genotyping of immune-related susceptibility loci reveals new insights into the genetics of psoriatic arthritis. Nat Commun.

[23] Coenen MJ, Trynka G, Heskamp S, Franke B, van Diemen CC, Smolonska J (2009). Common and different genetic background for rheumatoid arthritis and coeliac disease. Hum Mol Genet.

[24] Graham RR, Cotsapas C, Davies L, Hackett R, Lessard CJ, Leon JM (2008). Genetic variants near TNFAIP3 on 6q23 are associated with systemic lupus erythematosus. Nat Genet.

[25] Jostins L, Ripke S, Weersma RK, Duerr RH, McGovern DP, Hui KY (2012). Host-microbe interactions have shaped the genetic architecture of inflammatory bowel disease. Nature.

[26] Nair RP, Duffin KC, Helms C, Ding J, Stuart PE, Goldgar D (2009). Genome-wide scan reveals association of psoriasis with IL-23 and NF-kappaB pathways. Nat Genet.

[27] Onengut-Gumuscu S, Chen WM, Burren O, Cooper NJ, Quinlan AR, Mychaleckyj JC (2015). Fine mapping of type 1 diabetes susceptibility loci and evidence for colocalization of causal variants with lymphoid gene enhancers. Nat Genet.

[28] Thomson W, Barton A, Ke X, Eyre S, Hinks A, Bowes J (2007). Rheumatoid arthritis association at 6q23. Nat Genet.

[29] Wellcome Trust Case Control Consortium (2007). Genome-wide association study of 14,000 cases of seven common diseases and 3,000 shared controls. Nature.

[30] Adrianto I, Wen F, Templeton A, Wiley G, King JB, Lessard CJ (2011). Association of a functional variant downstream of TNFAIP3 with systemic lupus erythematosus. Nat Genet.

[31] Musone SL, Taylor KE, Lu TT, Nititham J, Ferreira RC, Ortmann W (2008). Multiple polymorphisms in the TNFAIP3 region are independently associated with systemic lupus erythematosus. Nat Genet.

[32] Ward LD, Kellis M (2012). HaploReg: a resource for exploring chromatin states, conservation, and regulatory motif alterations within sets of genetically linked variants. Nucleic Acids Res.

[33] Boyle AP, Hong EL, Hariharan M, Cheng Y, Schaub MA, Kasowski M (2012). Annotation of functional variation in personal genomes using RegulomeDB. Genome Res.

[34] Elsby LM, Orozco G, Denton J, Worthington J, Ray DW, Donn RP (2010). Functional evaluation of TNFAIP3 (A20) in rheumatoid arthritis. Clin Exp Rheumatol.

[35] Huber LC, Distler O, Tarner I, Gay RE, Gay S, Pap T (2006). Synovial fibroblasts: key players in rheumatoid arthritis. Rheumatology (Oxford).

[36] Hayden MS, West AP, Ghosh S (2006). NF-kappaB and the immune response. Oncogene.

[37] Bours V, Franzoso G, Azarenko V, Park S, Kanno T, Brown K (1993). The oncoprotein Bcl-3 directly transactivates through kappa B motifs via association with DNA-binding p50B homodimers. Cell.

[38] Carmody RJ, Ruan Q, Palmer S, Hilliard B, Chen YH (2007). Negative regulation of toll-like receptor signaling by NF-kappaB p50 ubiquitination blockade. Science.

[39] Wulczyn FG, Naumann M, Scheidereit C (1992). Candidate proto-oncogene bcl-3 encodes a subunit-specific inhibitor of transcription factor NF-kappa B. Nature.

[40] Schoenfelder S, Clay I, Fraser P (2010). The transcriptional interactome: gene expression in 3D. Curr Opin Genet Dev.

[41] Trynka G, Sandor C, Han B, Xu H, Stranger BE, Liu XS (2013). Chromatin marks identify critical cell types for fine mapping complex trait variants. Nat Genet.

[42] Rao SS, Huntley MH, Durand NC, Stamenova EK, Bochkov ID, Robinson JT (2014). A 3D map of the human genome at kilobase resolution reveals principles of chromatin looping. Cell.

[43] Pestka S, Krause CD, Sarkar D, Walter MR, Shi Y, Fisher PB (2004). Interleukin-10 and related cytokines and receptors. Annu Rev Immunol.

[44] Rutz S, Wang X, Ouyang W (2014). The IL-20 subfamily of cytokines--from host defence to tissue homeostasis. Nat Rev Immunol.

[45] Su AI, Wiltshire T, Batalov S, Lapp H, Ching KA, Block D (2004). A gene atlas of the mouse and human protein-encoding transcriptomes. Proc Natl Acad Sci U S A.

[46] Corvaisier M, Delneste Y, Jeanvoine H, Preisser L, Blanchard S, Garo E (2012). IL-26 is overexpressed in rheumatoid arthritis and induces proinflammatory cytokine production and Th17 cell generation. PLoS Biol.

[47] Hsu YH, Li HH, Hsieh MY, Liu MF, Huang KY, Chin LS (2006). Function of interleukin-20 as a proinflammatory molecule in rheumatoid and experimental arthritis. Arthritis Rheum.

[48] Ikeuchi H, Kuroiwa T, Hiramatsu N, Kaneko Y, Hiromura K, Ueki K (2005). Expression of interleukin-22 in rheumatoid arthritis: potential role as a proinflammatory cytokine. Arthritis Rheum.

[49] Kragstrup TW, Otkjaer K, Holm C, Jorgensen A, Hokland M, Iversen L (2008). The expression of IL-20 and IL-24 and their shared receptors are increased in rheumatoid arthritis and spondyloarthropathy. Cytokine.

[50] Sakurai N, Kuroiwa T, Ikeuchi H, Hiramatsu N, Maeshima A, Kaneko Y (2008). Expression of IL-19 and its receptors in RA: potential role for synovial hyperplasia formation. Rheumatology (Oxford).

[51] Hsu YH, Hsieh PP, Chang MS (2012). Interleukin-19 blockade attenuates collagen-induced arthritis in rats. Rheumatology (Oxford).

[52] Ouyang W, Rutz S, Crellin NK, Valdez PA, Hymowitz SG (2011). Regulation and functions of the IL-10 family of cytokines in inflammation and disease. Annu Rev Immunol.

[53] Blumberg H, Conklin D, Xu WF, Grossmann A, Brender T, Carollo S (2001). Interleukin 20: discovery, receptor identification, and role in epidermal function. Cell.

[54] He M, Liang P (2010). IL-24 transgenic mice: in vivo evidence of overlapping functions for IL-20, IL-22, and IL-24 in the epidermis. J Immunol.

[55] Wolk K, Haugen HS, Xu W, Witte E, Waggie K, Anderson M (2009). IL-22 and IL-20 are key mediators of the epidermal alterations in psoriasis while IL-17 and IFN-gamma are not. J Mol Med (Berl).

[56] Otkjaer K, Kragballe K, Funding AT, Clausen JT, Noerby PL, Steiniche T (2005). The dynamics of gene expression of interleukin-19 and interleukin-20 and their receptors in psoriasis. Br J Dermatol.

[57] Romer J, Hasselager E, Norby PL, Steiniche T, Thorn CJ, Kragballe K (2003). Epidermal overexpression of interleukin-19 and −20 mRNA in psoriatic skin disappears after short-term treatment with cyclosporine a or calcipotriol. J Invest Dermatol.

[58] Wolk K, Kunz S, Witte E, Friedrich M, Asadullah K, Sabat R (2004). IL-22 increases the innate immunity of tissues. Immunity.

[59] Gottlieb AB, Krueger JG, Sandberg LM, Gothberg M, Skolnick BE (2015). First-in-human, phase 1, randomized, dose-escalation trial with recombinant anti-il-20 monoclonal antibody in patients with psoriasis. PLoS One.

[60] Senolt L, Leszczynski P, Dokoupilova E, Gothberg M, Valencia X, Hansen BB (2015). Efficacy and safety of anti-interleukin-20 monoclonal antibody in patients with rheumatoid arthritis: a randomized phase IIa trial. Arthritis Rheumatol.

[61] Andoh A, Shioya M, Nishida A, Bamba S, Tsujikawa T, Kim-Mitsuyama S (2009). Expression of IL-24, an activator of the JAK1/STAT3/SOCS3 cascade, is enhanced in inflammatory bowel disease. J Immunol.

[62] Dambacher J, Beigel F, Zitzmann K, De Toni EN, Goke B, Diepolder HM (2009). The role of the novel Th17 cytokine IL-26 in intestinal inflammation. Gut.

[63] Fonseca-Camarillo G, Furuzawa-Carballeda J, Llorente L, Yamamoto-Furusho JK (2013). IL-10-- and IL-20--expressing epithelial and inflammatory cells are increased in patients with ulcerative colitis. J Clin Immunol.

[64] Fonseca-Camarillo G, Furuzawa-Carballeda J, Granados J, Yamamoto-Furusho JK (2014). Expression of interleukin (IL)-19 and IL-24 in inflammatory bowel disease patients: a cross-sectional study. Clin Exp Immunol.

[65] Nelson MR, Tipney H, Painter JL, Shen J, Nicoletti P, Shen Y (2015). The support of human genetic evidence for approved drug indications. Nat Genet.

[66] Hu X, Ivashkiv LB (2009). Cross-regulation of signaling pathways by interferon-gamma: implications for immune responses and autoimmune diseases. Immunity.

[67] Tang Q, Danila MI, Cui X, Parks L, Baker BJ, Reynolds RJ (2015). Expression of interferon-gamma receptor genes in peripheral blood mononuclear cells is associated with rheumatoid arthritis and its radiographic severity in African Americans. Arthritis Rheumatol.

[68] Barreiro LB, Tailleux L, Pai AA, Gicquel B, Marioni JC, Gilad Y (2012). Deciphering the genetic architecture of variation in the immune response to Mycobacterium tuberculosis infection. Proc Natl Acad Sci U S A.

[69] Fairfax BP, Makino S, Radhakrishnan J, Plant K, Leslie S, Dilthey A (2012). Genetics of gene expression in primary immune cells identifies cell type-specific master regulators and roles of HLA alleles. Nat Genet.

[70] Hu X, Kim H, Raj T, Brennan PJ, Trynka G, Teslovich N (2014). Regulation of gene expression in autoimmune disease loci and the genetic basis of proliferation in CD4+ effector memory T cells. PLoS Genet.

[71] Lee MN, Ye C, Villani AC, Raj T, Li W, Eisenhaure TM (2014). Common genetic variants modulate pathogen-sensing responses in human dendritic cells. Science.

[72] Romanoski CE, Lee S, Kim MJ, Ingram-Drake L, Plaisier CL, Yordanova R (2010). Systems genetics analysis of gene-by-environment interactions in human cells. Am J Hum Genet.

[73] Catrysse L, Vereecke L, Beyaert R (2014). van LG. A20 in inflammation and autoimmunity. Trends Immunol.

[74] Lee EG, Boone DL, Chai S, Libby SL, Chien M, Lodolce JP (2000). Failure to regulate TNF-induced NF-kappaB and cell death responses in A20-deficient mice. Science.

[75] Kilpinen H, Waszak SM, Gschwind AR, Raghav SK, Witwicki RM, Orioli A (2013). Coordinated effects of sequence variation on DNA binding, chromatin structure, and transcription. Science.

[76] Dixon JR, Selvaraj S, Yue F, Kim A, Li Y, Shen Y (2012). Topological domains in mammalian genomes identified by analysis of chromatin interactions. Nature.

[77] Pombo A, Dillon N (2015). Three-dimensional genome architecture: players and mechanisms. Nat Rev Mol Cell Biol.

[78] Kim YJ, Cecchini KR, Kim TH (2011). Conserved, developmentally regulated mechanism couples chromosomal looping and heterochromatin barrier activity at the homeobox gene A locus. Proc Natl Acad Sci U S A.

[79] Duggal G, Wang H, Kingsford C (2014). Higher-order chromatin domains link eQTLs with the expression of far-away genes. Nucleic Acids Res.

[80] Lupianez DG, Kraft K, Heinrich V, Krawitz P, Brancati F, Klopocki E (2015). Disruptions of topological chromatin domains cause pathogenic rewiring of gene-enhancer interactions. Cell.

[81] Eyre S, Bowes J, Diogo D, Lee A, Barton A, Martin P (2012). High-density genetic mapping identifies new susceptibility loci for rheumatoid arthritis. Nat Genet.

[82] Hinks A, Cobb J, Marion MC, Prahalad S, Sudman M, Bowes J (2013). Dense genotyping of immune-related disease regions identifies 14 new susceptibility loci for juvenile idiopathic arthritis. Nat Genet.

[83] Okada Y, Wu D, Trynka G, Raj T, Terao C, Ikari K (2014). Genetics of rheumatoid arthritis contributes to biology and drug discovery. Nature.

[84] Hughes JR, Roberts N, McGowan S, Hay D, Giannoulatou E, Lynch M (2014). Analysis of hundreds of cis-regulatory landscapes at high resolution in a single, high-throughput experiment. Nat Genet.

[85] Belton JM, McCord RP, Gibcus JH, Naumova N, Zhan Y, Dekker J (2012). Hi-C: a comprehensive technique to capture the conformation of genomes. Methods.

[86] van Berkum NL, Lieberman-Aiden E, Williams L, Imakaev M, Gnirke A, Mirny LA, et al. Hi-C: a method to study the three-dimensional architecture of genomes. J Vis Exp. 2010. DOI: 10.3791/1869. 10.3791/1869 PMC3149993 20461051

[87] Bolger AM, Lohse M, Usadel B (2014). Trimmomatic: a flexible trimmer for Illumina sequence data. Bioinformatics.

[88] Naumova N, Smith EM, Zhan Y, Dekker J (2012). Analysis of long-range chromatin interactions using Chromosome Conformation Capture. Methods.

[89] Zhou X, Maricque B, Xie M, Li D, Sundaram V, Martin EA (2011). The Human Epigenome Browser at Washington University. Nat Methods.

[90] Zhou X, Lowdon RF, Li D, Lawson HA, Madden PA, Costello JF (2013). Exploring long-range genome interactions using the WashU Epigenome Browser. Nat Methods.

[91] Montgomery SB, Sammeth M, Gutierrez-Arcelus M, Lach RP, Ingle C, Nisbett J (2010). Transcriptome genetics using second generation sequencing in a Caucasian population. Nature.

[92] Stranger BE, Nica AC, Forrest MS, Dimas A, Bird CP, Beazley C (2007). Population genomics of human gene expression. Nat Genet.

[93] Schadt EE, Molony C, Chudin E, Hao K, Yang X, Lum PY (2008). Mapping the genetic architecture of gene expression in human liver. PLoS Biol.

[94] Gibbs JR, van der Brug MP, Hernandez DG, Traynor BJ, Nalls MA, Lai SL (2010). Abundant quantitative trait loci exist for DNA methylation and gene expression in human brain. PLoS Genet.

[95] Li Q, Stram A, Chen C, Kar S, Gayther S, Pharoah P (2014). Expression QTL-based analyses reveal candidate causal genes and loci across five tumor types. Hum Mol Genet.

[96] Westra HJ, Peters MJ, Esko T, Yaghootkar H, Schurmann C, Kettunen J (2013). Systematic identification of trans eQTLs as putative drivers of known disease associations. Nat Genet.

[97] Hao K, Bosse Y, Nickle DC, Pare PD, Postma DS, Laviolette M (2012). Lung eQTLs to help reveal the molecular underpinnings of asthma. PLoS Genet.

[98] Koopmann TT, Adriaens ME, Moerland PD, Marsman RF, Westerveld ML, Lal S (2014). Genome-wide identification of expression quantitative trait loci (eQTLs) in human heart. PLoS One.

[99] Grundberg E, Adoue V, Kwan T, Ge B, Duan QL, Lam KC (2011). Global analysis of the impact of environmental perturbation on cis-regulation of gene expression. PLoS Genet.

[100] Lappalainen T, Sammeth M, Friedlander MR, ‘t Hoen PA, Monlong J, Rivas MA (2013). Transcriptome and genome sequencing uncovers functional variation in humans. Nature.

[101] Ramasamy A, Trabzuni D, Guelfi S, Varghese V, Smith C, Walker R (2014). Genetic variability in the regulation of gene expression in ten regions of the human brain. Nat Neurosci.

[102] Ritchie ME, Phipson B, Wu D, Hu Y, Law CW, Shi W (2015). limma powers differential expression analyses for RNA-sequencing and microarray studies. Nucleic Acids Res.

