# Supplementary material for: Capture Hi-C identifies a novel causal gene, IL20RA, in the pan-autoimmune genetic susceptibility region 6q23
Source: Genome Biol. 2016 Nov 1;17:212. doi: 10.1186/s13059-016-1078-x (PMC5088679; doi:10.1186/s13059-016-1078-x)

**Additional file 1**

**Supplemental information**

**Table S1.** RegulomeDB functional annotation of intergenic 6q23 SNPs associated with autoimmunity. 2b: likely to affect binding; 3a; less likely to affect binding; 6: minimal binding evidence.

| **dbSNP ID** | **LD with lead SNP (r^2^)** | **Regulome DB Score** |
| --- | --- | --- |
| rs6927172 | - | 2b |
| rs35926684 | 0.86 | 3a |
| rs62432712 | 0.96 | 6 |
| rs2327832 | 0.95 | 6 |
| rs928722 | 0.95 | 6 |
| rs17264332 | 1 | 6 |
| rs6920220 | 1 | 6 |
| rs6933404 | 0.96 | No Data |

**Table S2.** B lymphoblastoid cell lines used.

| ID | rs6927172 genotype |
| --- | --- |
| GM10838 | CC |
| GM10843 | CC |
| GM07056 | CC |
| GM10848 | CC |
| GM11993 | CC |
| GM10860 | CC |
| GM12056 | CC |
| GM12707 | CC |
| GM06985 | CC |
| GM12892 | CC |
| GM12878 | CG |
| GM11994 | CG |
| GM12145 | CG |
| GM12812 | CG |
| GM12875 | CG |
| GM10831 | CG |
| GM06993 | CG |
| GM10858 | GG |
| GM10850 | GG |
| GM12560 | GG |

**Table S3.** BAC Clones used for 3C validation.

| **BAC clone ID** | **Start position** | **End Position** | **Size** |  |
| --- | --- | --- | --- | --- |
| RP11-162O9 | Chr6: 137286536 | Chr6: 137450559 | 164024 (+ strand) |  |
| RPCI-11 1058B18 | Chr6: 137425741 | Chr6: 137630056 | 204316 (- strand) |  |
| HS-CITB 2071P12 | Chr6: 137625360 | Chr6: 137804064 | 178705 (+ strand) |  |
| HS-CITB 3175H21 | Chr6: 137799448 | Chr6: 137879516 | 80069 (- strand) |  |
| HS-CITB 2374P13 | Chr6: 137867732 | Chr6: 137992452 | 124721 (- strand) |  |
| HS-CITB 2511N24 | Chr6: 137916906 | Chr6: 138137421 | 220516 (- strand) |  |
| HS-CITB 2244P2 | Chr6: 138128388 | Chr6: 138273180 | 144793 (- strand) |  |
| HS-CITB 2106E2 | Chr6: 138268647 | Chr6: 138400874 | 132228 (- strand) |  |
| RPCI-11 1023E5 | Chr6: 138393699 | Chr6: 138591433 | 197735 (- strand) |  |

**Table S4.** 3C-qPCR primers.

| **Primer** | **Sequence** |
| --- | --- |
| IL20RA_1_B | TCCGAAGAGCTTTGTTTGTGG |
| IL20RA_2_B | TGCTGCCCAGACATAGGAAA |
| IL20RA_3_B | AATGCGACTGTCAAGGATGC |
| IL20RA_3_C | TGCCATTCTAGCCCTTCCAG |
| IFNGR1_1 | CAAGGCAAGGTGGTGGTTTT |
| SNPs_1_A | GCTGATTGTGGAAGAGTTCAGT |
| SNPs_1_B | AGACAGACTTGAGTGCCTATTG |
| SNPs_2_B | CCAGCAGGCAGAGAAAGAAT |
| SNPs_3 | CTGACTTTGTGATCCGCCTG |
| SNPs_5_B | GTCCCACCTCTGTCCAAAGA |
| psPTPN11_1 | ATCCCACCTGGCTGTCTATG |
| Y_RNA | TCCATATCCCGTCAGCACAA |
| TNFAIP3_4_B | GGCTTTGGAGTAACACAGGC |
| TNFAIP3_2_B | AGCCCTCATCGACAGAAACA |
| TNFAIP3_1_B | TCTGTGCTGTTCTGCCAATG |
| TNFAIP3_3_B | AGGAAAGGGATGCTAGGACC |
| lncRNA_4_A | AGTTCGTTTCTCCCTGGGTT |
| lncRNA_4_B | AGTCTAGCTGGTTTGGGAGG |
| lncRNA_1_B | TCAGACTGTGGAGCTTGAGG |
| lncRNA_3 | CTAAAGCAGACCAAGCCACC |
| lncRNA_2_A | TGGACAGATTGGCAGGAAGA |
| Downstream | GCCGAAATGCCTGCTATGTT |
| NCR _A1 | ACAGGCAGTGGTATGTTGGA |
| NCR _B1 | GGGGCTCAGTGTTCTCAGAT |
| P_3 | ATGGTTCTGCAAGGCTGTG |
| P_4 | GTTGTCTGCCTCTGGATCCC |
| P_6 | CAGGTGTGAGCCATAATGCC |
| P_7 | GAGCAGGAAATGGAGGGAGG |
| P_10 | GGCTTTGGAGTAACACAGGC |

**Table S5.** ChIP-qPCR primers. Target Primers were designed to exclude the SNP region from the design to obtain flanking primers. Positive control primers were designed using an NF-κB binding site (-316/-15) in the human IkBα gene promoter.

| **Primer** | **Sequence** |
| --- | --- |
| **NF-κB** |  |
| Positive_1_F | GGAATTTCCAAGCCAGTCAG |
| Positive_1_R | CTCATCGCAGGGAGTTTCTC |
| Neg_1_F | TGGGCAACAAGAATGAGACC |
| Neg_1_R | TTAAAGGCAGGGGATGAGTG |
| Neg_2_F | TGAGAATCACCAGGGCTCTT |
| Neg_2_R | CCACTGGGGTGACTCTTAGC |
| **H3K4me1** |  |
| POS coGAPDH_FWD | TAGAGGGGTGATGTGGGGAG |
| POS coGAPDH_REV | AGTGATGGCATGGACTGTGG |
| Neg_1_FWD | CTGAAGGGATCTGGCACAGT |
| Neg_1_REV | CCAACTGGCCAGGTAGTGAT |
| **H3K27Ac** |  |
| Neg_3_FWD | ACTCATAACTCCCAGGTGCG |
| Neg_3_REV | CGAAGGCCAAAAACTGGTCC |
| Pos_3_FWD | CACTTGCAGAGGGACAGGAT |
| Pos_3_REV | GAGAAACTCCCTGCGATGAG |
| **Target** |  |
| rs6927172 Target_1_F | GGAGCTAATCAAGTGGCAATG |
| rs6927172 Target_1_R | TGATTTCTCCCTGAGGTCAGTT |

**Figure S1.** Long-range interactions in the 6q23 locus analyzed with CHiCAGO. Genomic co-ordinates are shown along the top of each panel and tracks are labelled A-N: A – HindIII restriction fragments; B-E – Regions targeted and restriction fragments included in the Region (B, C) and Promoter (D, E) Capture experiments; F – GENCODE V17 genes; G, H, I, –1000 Genomes SNPs in LD (r2≥0.8) with the index SNPs rs6920220, associated with RA, SLE, celiac disease, T1D and IBD (G), rs7752903, associated with RA, SLE and celiac disease (H) and rs610604, associated with Ps and PsA (I); J- Topologically associated domains (TADs) in GM12878 cells *(20)* ; K-N – Significant Interactions identified in the Region and Promoter capture experiments in GM12878 (K, L) and Jurkat (M, N) cells.

**
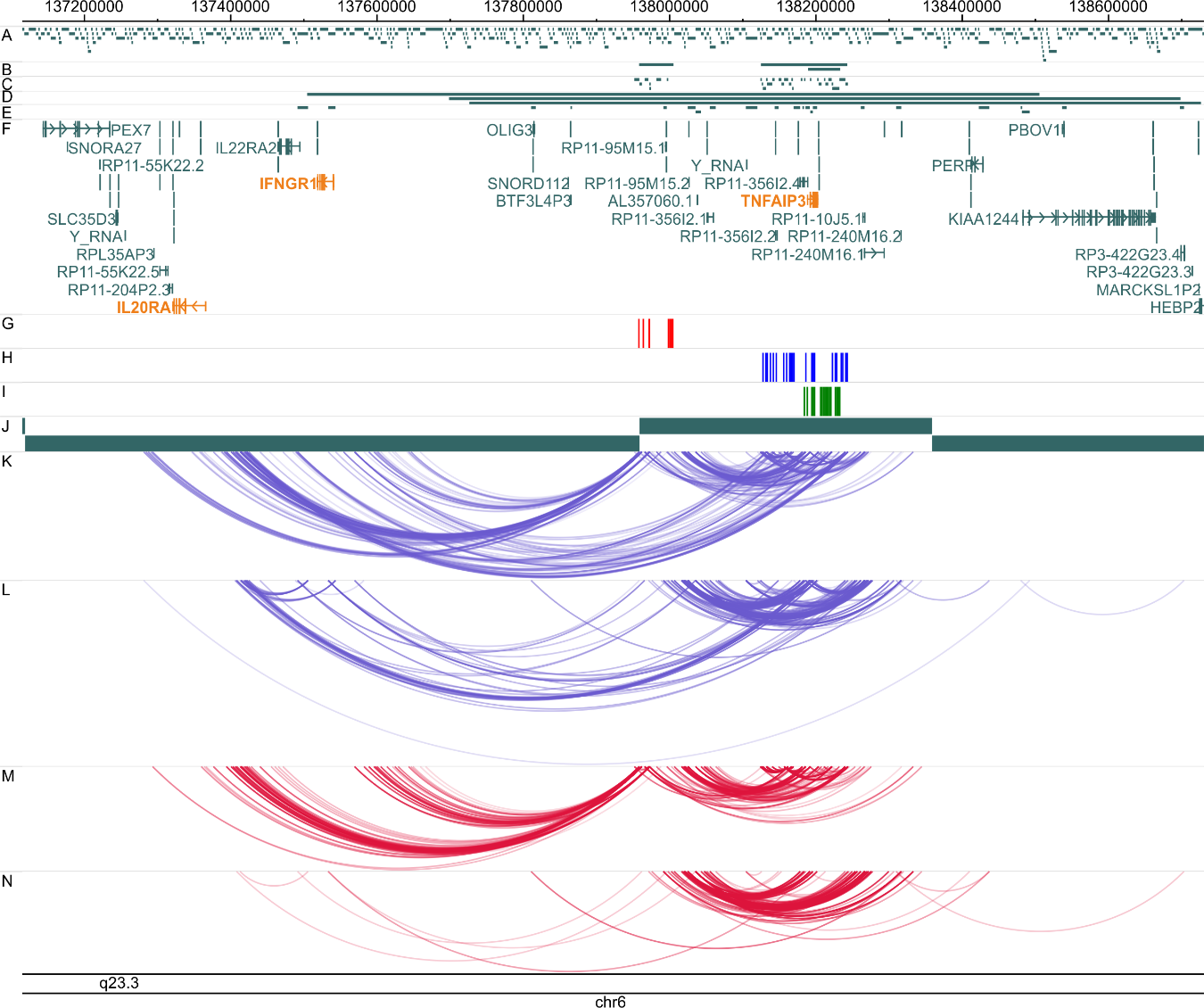
**

**Figure S2.** Genotype-specific 3C in LCLs showing that interaction frequencies between the fragment containing rs6927172 and fragments containing the lncRNAs RP11-10J5.1 (A) and RP11-240M16.1 (B) were similar regardless of genotype; NCR, non-interacting control region. Error bars indicate standard deviation of three biological replicates.


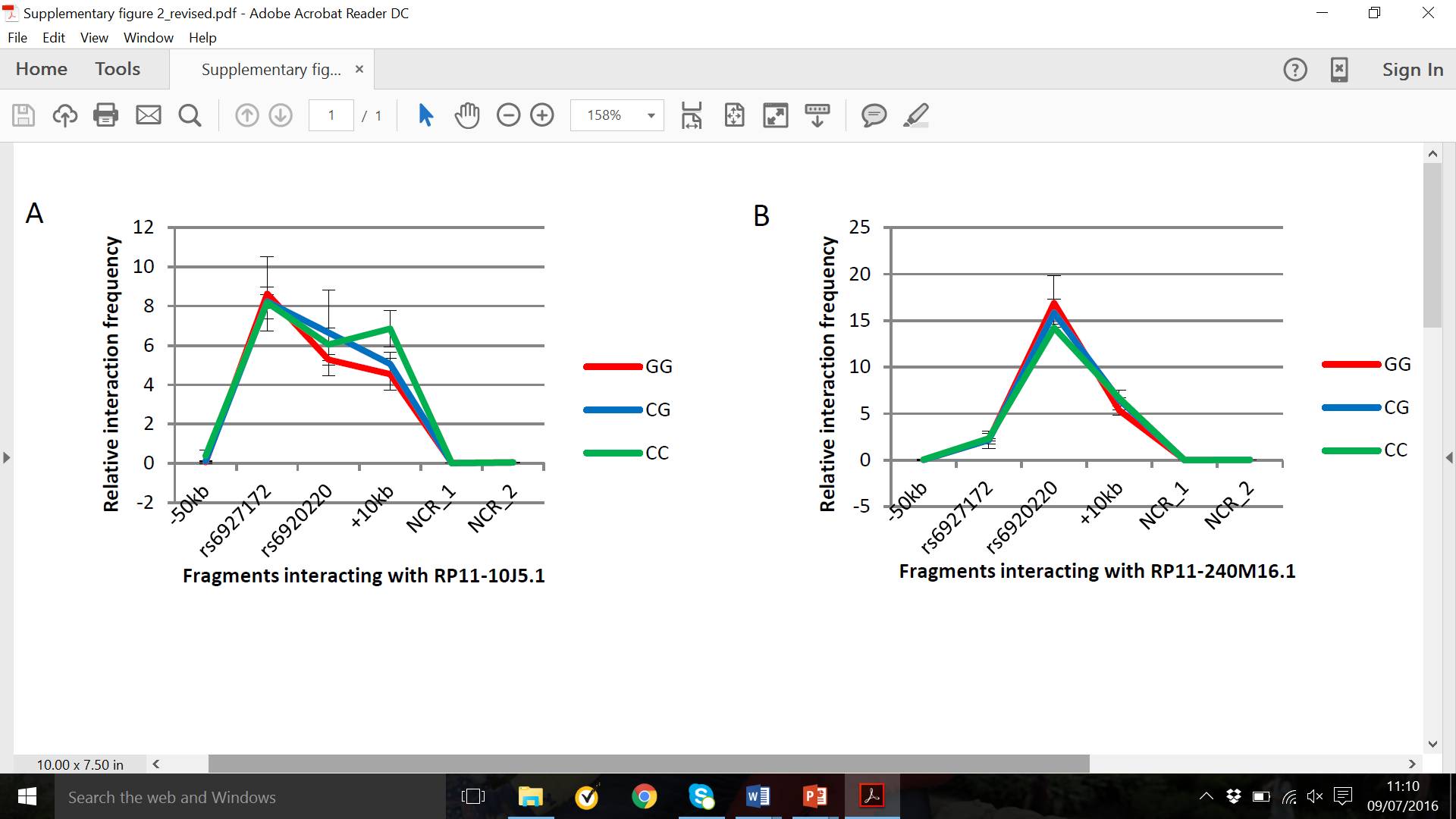


**Figure S3.** Genotype-specific 3C in LCLs showing that interactions between *TNFAIP3* and *PTPN11* pseudogene (A), RP11-10J5.1 (B), RP11-240M16.1 (C), Y_RNA (D) and *IL20RA* (E) and between *IL20RA* and RP11-10J5.1 (F) were not influenced by rs6927172 genotype; NCR, non-interacting control region. Error bars indicate standard deviation of three biological replicates.


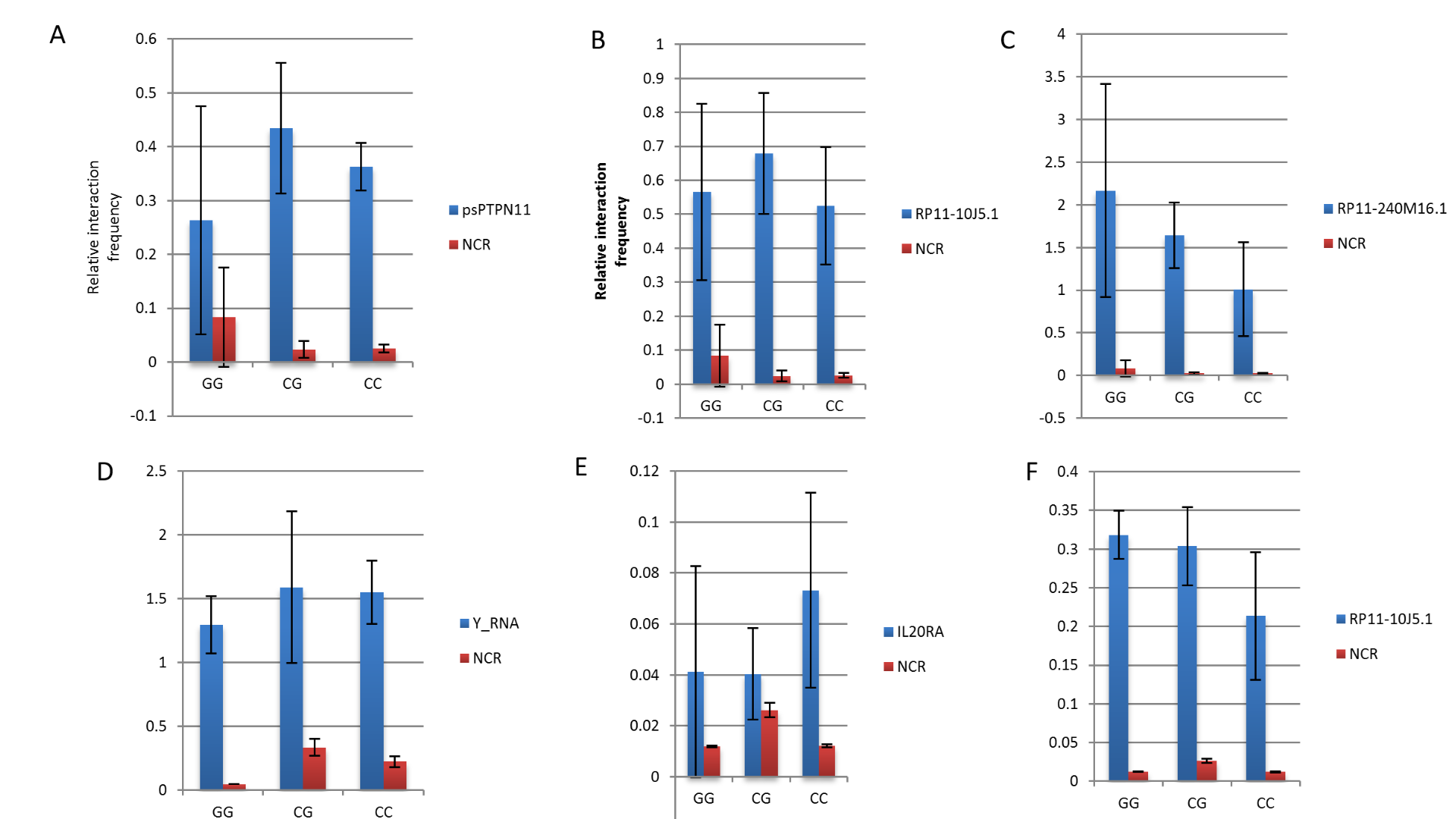


**Figure S4.** 3C showing strong interaction of the rs6927172 containing restriction fragment with *IL20RA* in primary synovial fibroblasts. -50kb, restriction fragment located 50 kb upstream of the rs6927172 containing restriction fragment; rs6927172: restriction fragment containing rs6927172; rs6920220, restriction fragment containing the top GWAS SNP in the 6q23 region; NCR, non-interacting control region. Error bars indicate standard deviation of three biological replicates

**
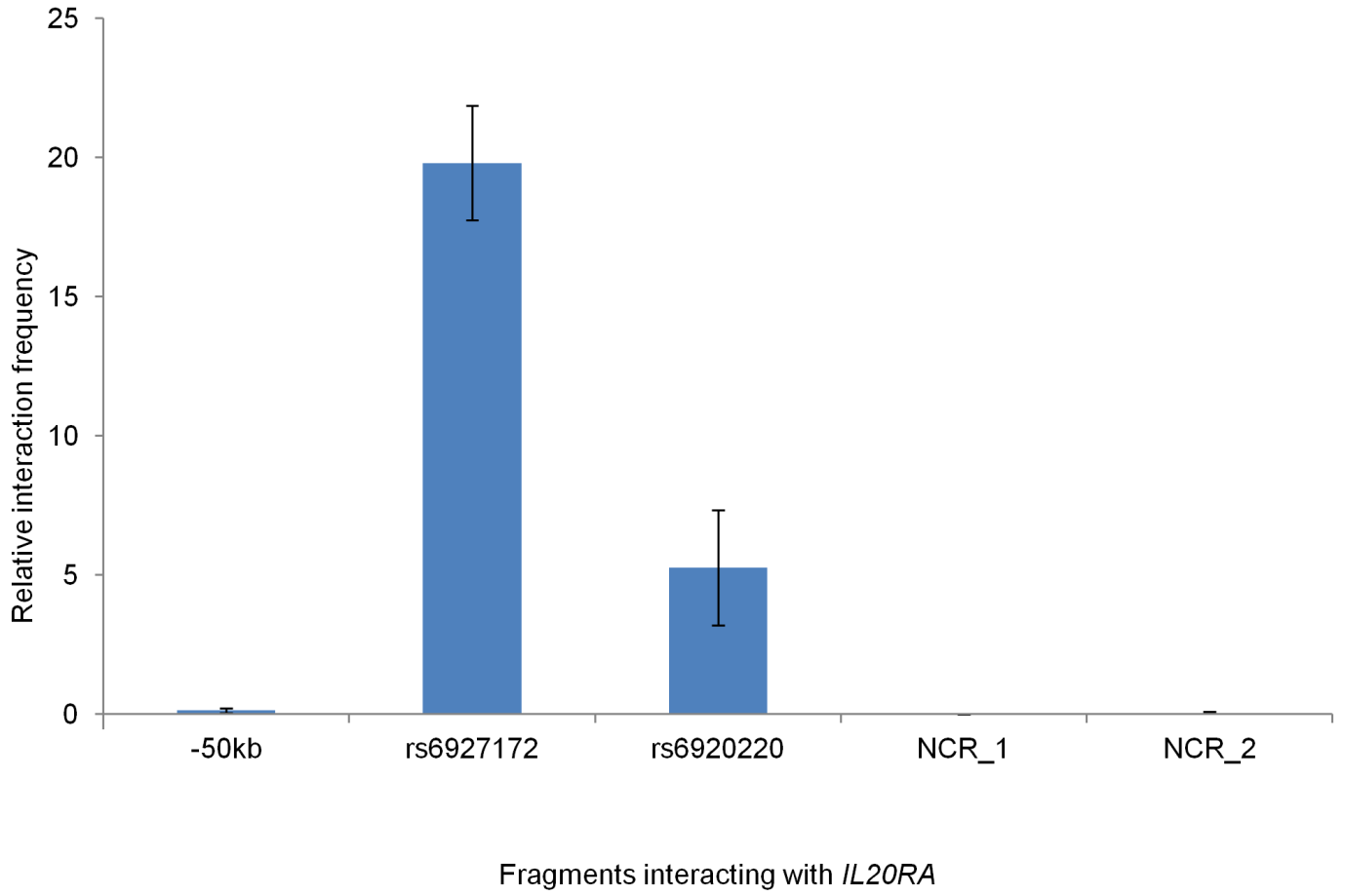
**

**Figure S5.** ChIP showing binding of the histone marks for active enhancers H3K4me1 and H3K27ac to the rs6927172 SNP. Error bars indicate standard deviation of 17 different B-lymphoblastoid cell lines.


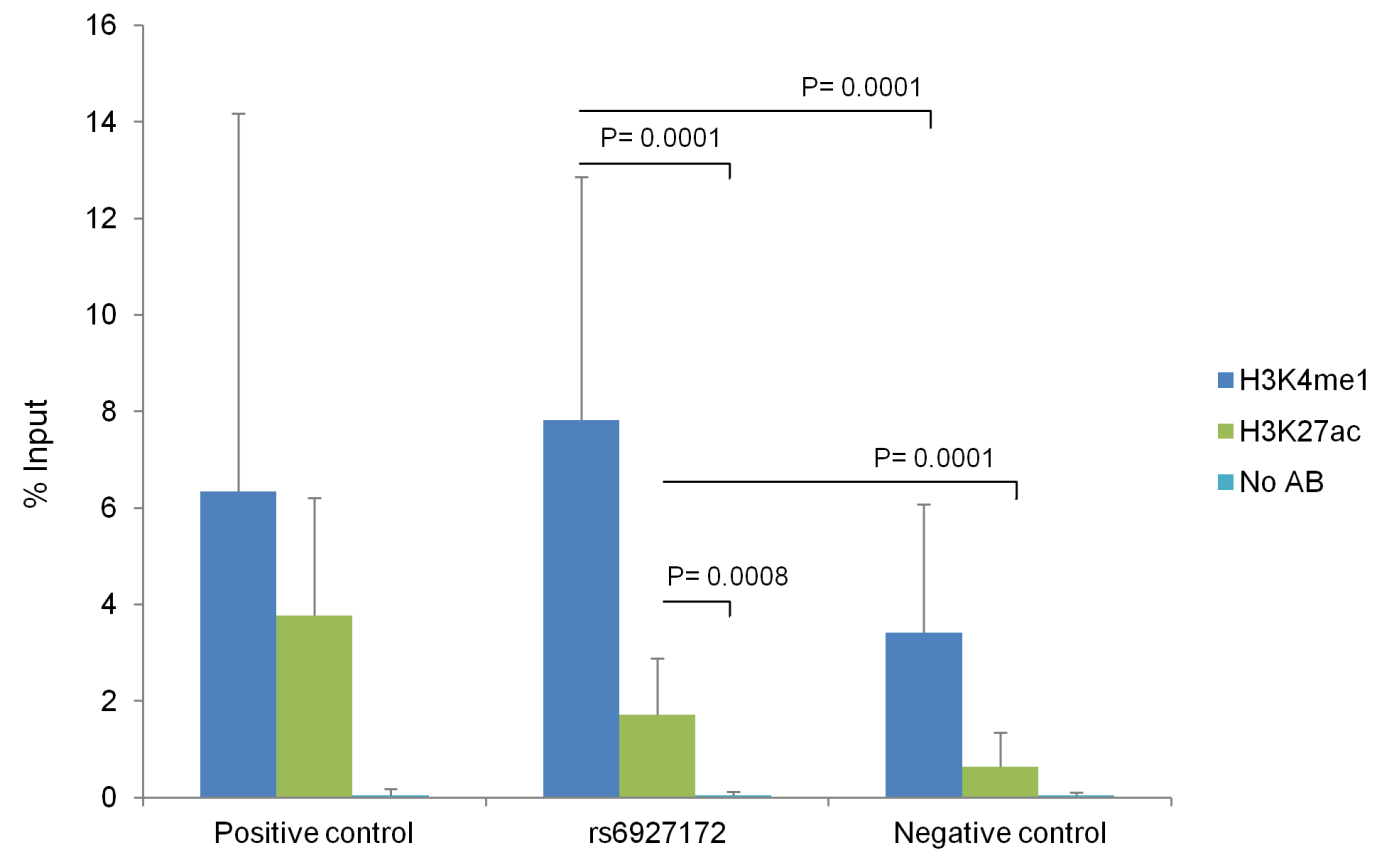


**Figure S6.** Allele specific ChIP in GM12145 cells. * p= 0.003, ** p=0.001.

**
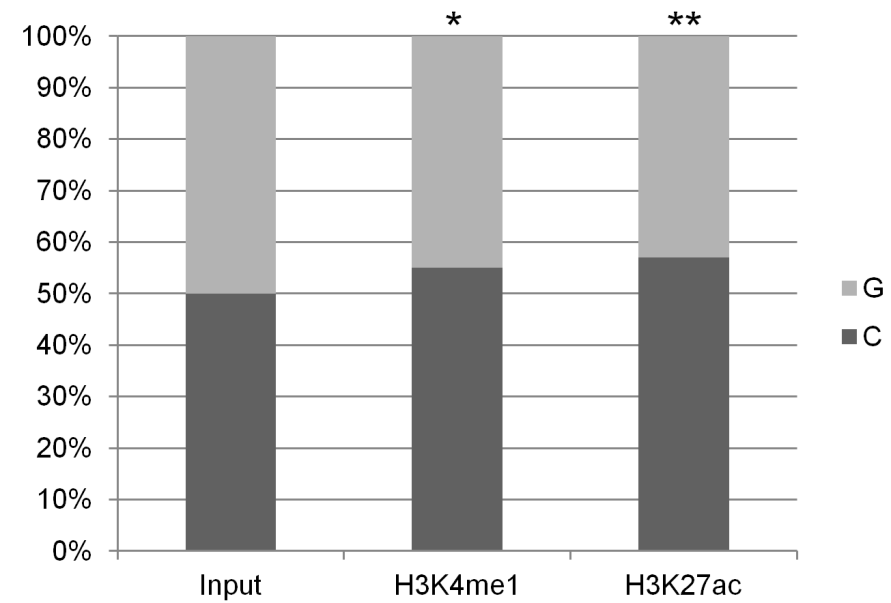
**

**Figure S7**. High resolution Hi-C data obtained from Rao et al [42] showing numerous, strong interactions between 6q23 intergenic SNPs tagged by rs6920220 and *IL20RA* in GM12878 B-lymphoblastoid cell line. In contrast, cells that do not express *IL20RA*, such as HUVEC and K562 present less or no interactions (observed/expected interactions >10).


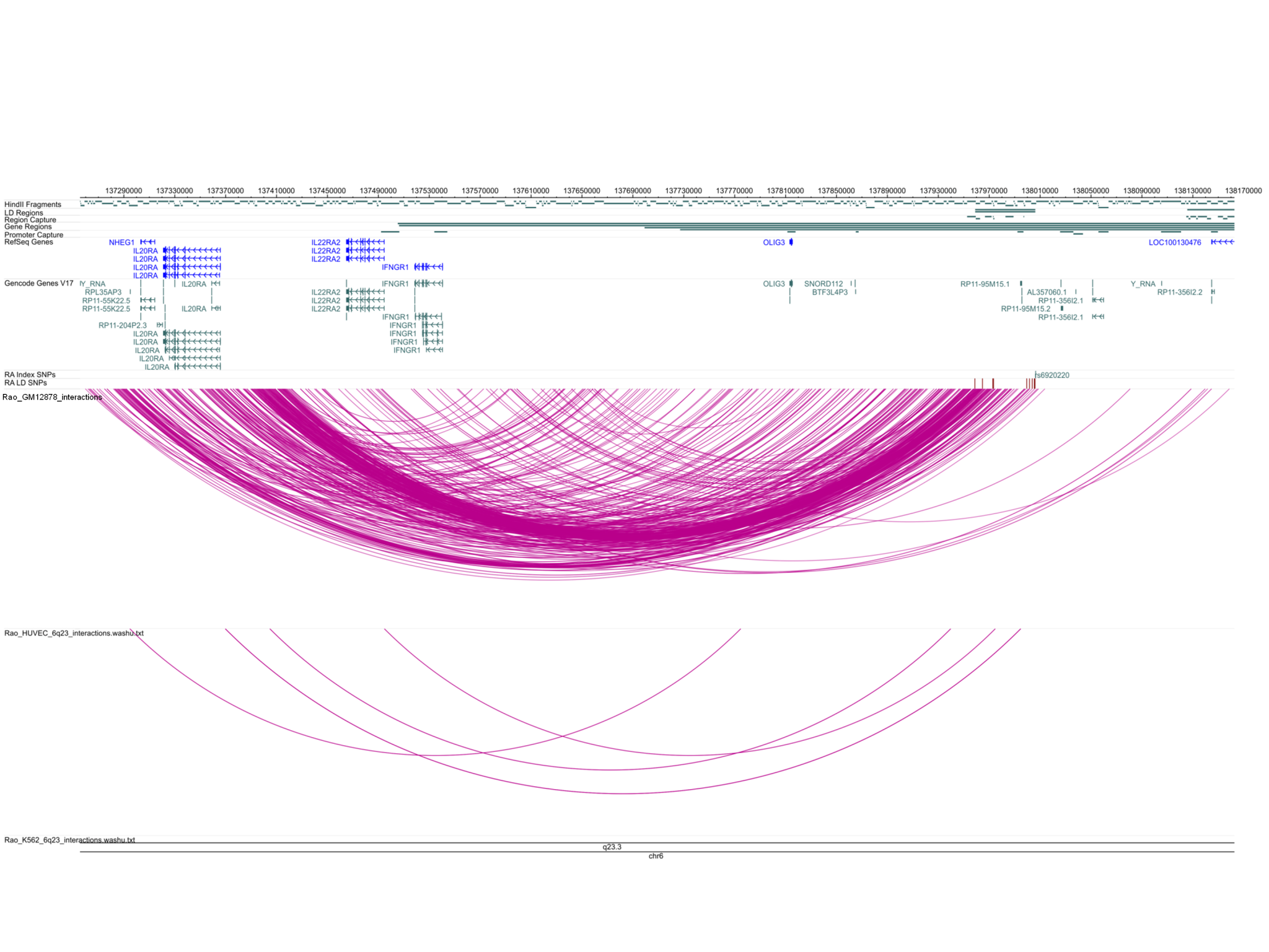

Supplement: Additional file 1: — Supplementary tables and figures. (DOCX 2089 kb) [file 13059_2016_1078_MOESM1_ESM.docx]
